# Aging alters mechanisms underlying voluntary movements in spinal motor neurons of mice, primates, and humans

**DOI:** 10.1172/jci.insight.168448

**Published:** 2023-05-08

**Authors:** Ryan W. Castro, Mikayla C. Lopes, Robert E. Settlage, Gregorio Valdez

**Affiliations:** 1 Neuroscience Graduate Program,; 2 Department of Molecular Biology, Cellular Biology, and Biochemistry,; 3 Center for Translational Neuroscience, Robert J. and Nancy D. Carney Institute for Brain Science and Brown Institute for Translational Science, and; 4 Molecular Biology, Cell Biology, and Biochemistry Graduate Program, Brown University, Providence, Rhode Island, USA.; 5 Department of Advanced Research Computing, Virginia Tech, Blacksburg, Virginia, USA.; 6 Department of Neurology, Warren Alpert Medical School of Brown University, Providence, Rhode Island, USA.

**Keywords:** Aging, Neuroscience, Movement disorders, Neurodegeneration, Neuromuscular disease

## Abstract

Spinal motor neurons have been implicated in the loss of motor function that occurs with advancing age. However, the cellular and molecular mechanisms that impair the function of these neurons during aging remain unknown. Here, we show that motor neurons do not die in old female and male mice, rhesus monkeys, and humans. Instead, these neurons selectively and progressively shed excitatory synaptic inputs throughout the soma and dendritic arbor during aging. Thus, aged motor neurons contain a motor circuitry with a reduced ratio of excitatory to inhibitory synapses that may be responsible for the diminished ability to activate motor neurons to commence movements. An examination of the motor neuron translatome (ribosomal transcripts) in male and female mice reveals genes and molecular pathways with roles in glia-mediated synaptic pruning, inflammation, axonal regeneration, and oxidative stress that are upregulated in aged motor neurons. Some of these genes and pathways are also found altered in motor neurons affected with amyotrophic lateral sclerosis (ALS) and responding to axotomy, demonstrating that aged motor neurons are under significant stress. Our findings show mechanisms altered in aged motor neurons that could serve as therapeutic targets to preserve motor function during aging.

## Graphical abstract

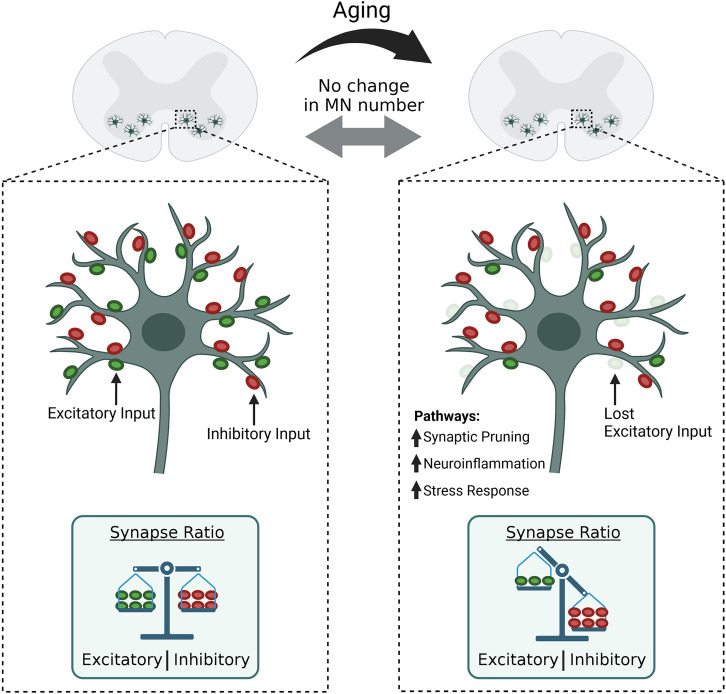

## Introduction

The ability to command voluntary movements progressively erodes during normal aging, characterized by deficits in gait speed, balance, and control of fine motor skills (1–3). In this regard, spinal cord motor neurons have been extensively studied because they are essential for voluntary movement (4–6). Spinal motor neurons are the largest neurons in the spinal cord with expansive dendritic arbors and long axons that connect with skeletal muscle fibers. Within the spinal cord, motor neurons form tens of thousands of excitatory (glutamatergic and cholinergic) and inhibitory (GABAergic and glycinergic) synapses (7) along their dendritic arbor and soma. These synapses contain the information required to execute fine and complex motor commands (8) and are together referred to as the motor circuitry. Once activated, motor neurons drive muscle contraction by releasing neurotransmitters from their axon terminals at neuromuscular junctions (NMJs) (9). In addition to integrating and relaying all motor commands through neurotransmission, motor neurons release molecules such as neuregulin-1, z-agrin, and brain-derived neurotrophic factor that are essential for the formation and maintenance of motor synapses (10).

A number of deleterious changes in motor neurons, particularly in their axons, have been linked to age-related impairments of motor function. Motor axons have been shown to lose the ability to transmit motor signals from the spinal cord to the NMJ with high fidelity. This is due to impaired propagation of action potentials (11), aberrant discharge of acetylcholine at the NMJ (12), redistribution or complete loss of active zones at the presynapse (13), and degeneration of axon terminals (14–18). In stark contrast to these changes in motor axons, the status of aged motor neurons within the spinal cord, both at a cellular and molecular level, is much less clear. For instance, there are varied reports on the extent to which aging impacts the number and size of motor neurons (14, 17, 19–33). A possible explanation for these findings may be differences in methods of quantifying motor neuron loss, including the use of indirect methods, such as counting motor axons in the ventral root or sciatic nerve or labeling motor neurons with nonspecific markers. Regardless, the question of whether motor neurons degenerate prior to loss of motor function with aging remains debated. There is even less known about the impact of aging on the motor dendritic arbor and the synapses it forms with other neurons in the central and peripheral nervous systems. Additionally, the molecular composition of aging motor neurons has yet to be defined. These major gaps in knowledge must be filled to identify the mechanisms by which motor neurons contribute to age-related motor deficits.

In this study, we used complementary approaches to identify the cellular and molecular features of aging spinal motor neurons in mice of both sexes, rhesus monkeys, and humans. We used multiple labeling methods, including IHC, RNAscope, and transgenic labeling, to ascertain the fate of aging motor neurons. We show that neither the number nor size of motor neurons are significantly affected by aging. The dendritic arbor of motor neurons is also largely unchanged with age. However, aging results in the selective loss of excitatory synaptic inputs from the soma and dendritic arbor of motor neurons. We then profiled the translatome of motor neurons using ribosome-associated mRNA to identify molecular features that are altered by aging. We demonstrate that aged motor neurons differentially express genes with roles in synaptic plasticity, immune activation, axonal regeneration, and oxidative stress. This aging transcriptional signature is largely shared by male and female mice. Additionally, aging motor neurons recruit similar axonal regeneration pathways as axotomized and amyotrophic lateral sclerosis–affected (ALS-affected) motor neurons. These data provide the framework to target specific cellular sites and molecular pathways to stave off the deleterious effects of aging on motor neurons in different species and in both sexes.

## Results

### Motor neuron number and size do not significantly change during aging.

**Figure 1:**
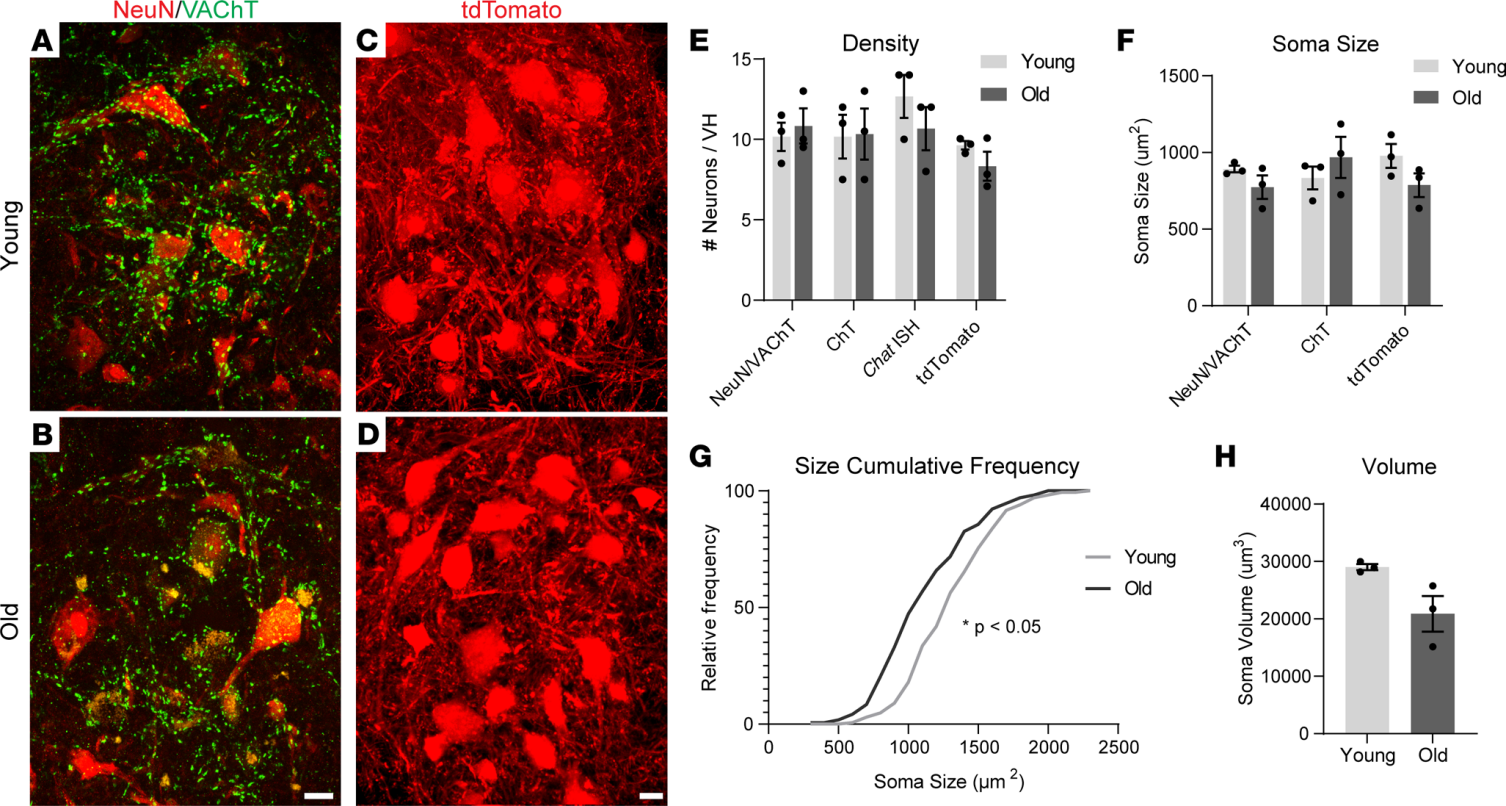
Motor neuron numbers and sizes are stable in aged spinal cords. (**A**–**D**) Representative images of α-motor neurons in young adult (top row) and aged (bottom row) mouse spinal cords. (**A** and **B**) NeuN and VAChT IHC in males. (**C** and **D**) Transgenic tdTomato labeled motor neurons in Chat-Cre;tdTomato males. (**E**) Motor neuron density in lumbar spinal cord of male mice based on multiple methods of α-motor neuron labeling, including NeuN and VAChT IHC, ChT IHC, *Chat* in situ hybridization, and transgenic tdTomato expression in Chat-Cre;tdTomato mice. (**F**) Cross-sectional area (CSA) of α-motor neuron soma based on NeuN and VAChT IHC, ChT IHC, and Chat-Cre;tdTomato labeling in male mouse lumbar spinal cord. (**G**) Distribution of α-motor neuron CSAs in male mouse lumbar spinal cord. (**H**) Volume of tdTomato-labeled α-motor neurons in male Chat-Cre;tdTomato lumbar spinal cord. Unpaired, 2-sided *t* test used for all comparisons except **G**, where a Kolmogorov-Smirnov test was used. *P < 0.05 versus young. All values except **G** presented as mean ± SEM; *n* = 3. Scale bar: 20 μm.

To ascertain the impact of aging on spinal motor neurons, we examined these neurons in aged male and female mice. We first measured the density of motor neuron soma in coronal sections of the lumbar spinal cord of 3 month and 22- to 24-month-old mice, an age at which motor function is in decline (34). We used multiple labeling techniques to ensure that motor neurons were specifically counted. Our techniques included IHC to identify large NeuN^+^ neuronal soma surrounded by vesicular acetylcholine transporter^+^ (VAChT^+^) synaptic puncta (Figure 1, A and B, and Supplemental Figure 1, E and F; supplemental material available online with this article; https://doi.org/10.1172/jci.insight.168448DS1) or choline transporter^+^ (ChT^+^) soma (Supplemental Figure 1, A and B) in the ventral horn; RNAscope to visualize choline acetyltransferase (*Chat*) mRNA^+^ soma (Supplemental Figure 1, C and D); and use of Chat-Cre;Ai14 mice, herein referred to as Chat-Cre;tdTomato mice, plus PACT-CLARITY tissue clearing to examine hundreds of tdTomato-labeled motor neurons (Figure 1, C and D). Using these complementary approaches, we found no difference in the density of motor neurons in the lumbar spinal cord in aged compared with young male (Figure 1E) or female (Supplemental Figure 1G) mice. We therefore conclude that loss of motor neurons is not a primary driver of age-related motor deficits in mice.

We then explored the possibility that motor neurons decrease in size with advancing age, as has been shown to occur during the progression of motor diseases (35). We measured the cross-sectional area (CSA) of motor neuron soma from young and aged mice using each of the labeling techniques described above. We found that the average motor neuron soma CSA was not significantly affected by age in female or male mice (Figure 1F and Supplemental Figure 1I). To gain additional insights, we examined the distribution of motor neuron CSA and volume from Chat-Cre;tdTomato mice, from which we imaged hundreds of motor neurons per animal. A cumulative frequency histogram showed a statistically significant shift toward smaller soma sizes in the aged group (Figure 1G). We also found a moderate but nonsignificant decrease in overall soma volume (Figure 1H). Additional analysis using a size histogram shows that the largest motor neurons are preferentially affected and, therefore, account for the size reduction in aged Chat-Cre;tdTomato mice (Supplemental Figure 1H). These findings show that, while the number of motor neurons remains unchanged in aged animals, the largest motor neuron subtypes, which are more susceptible to diseases (36), decrease in size.

### The dendritic tree does not degenerate in aged mice.

**Figure 2:**
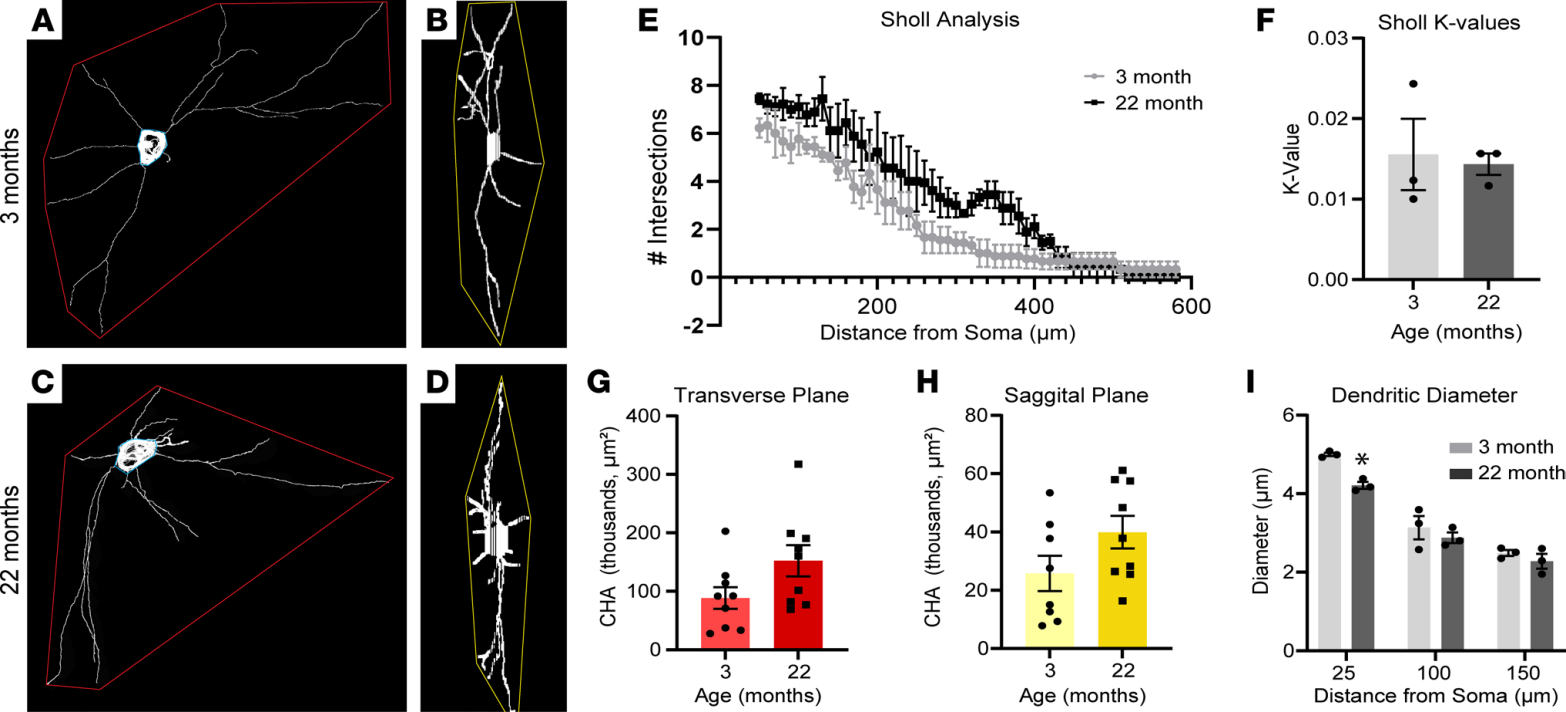
The dendritic arbor does not degenerate in aged mice. (**A** and **C**) Representative traces of tdTomato-labeled motor neuron dendritic arbors in transverse plane in young (3 month) and aged (22 month) Chat-Cre;tdTomato mouse lumbar spinal cord following PACT-CLARITY tissue clearing. (**B** and **D**) Representative dendritic arbor traces in sagittal plane. Convex hull areas (CHA) depicted by red or yellow outlines. (**E**) Sholl analysis of motor neuron dendritic arbor complexity. (**F**) Mean *K* values of dendritic arbor complexity Sholl analysis. (**G** and **H**) CHA of dendritic arbors measured in transverse (**G**) or sagittal (**H**) planes of spinal cords. Data points represent individual motor neurons from 3 animals per age group. (**I**) Motor neuron dendrite thickness at 25, 100, and 150 μm from the soma. Unpaired, 2-sided *t* test used for all comparisons. *P < 0.05 versus young. All values presented as mean ± SEM; *n* = 3.

We next examined the effect of aging on the dendritic arbor, which is where most motor signals are relayed to motor neurons (37–40). For this, we traced the dendritic arbor of motor neurons expressing tdTomato in 500 μm–thick coronal sections of lumbar spinal cords of young and aged Chat-Cre;tdTomato mice cleared using PACT-CLARITY (41). This approach made it possible to trace tdTomato-labeled dendrites at an average distance of 458 μm from the soma (Supplemental Video 1). Sholl analyses of traced dendrites did not reveal overt signs of dendritic arbor degeneration in aged mice (Figure 2, A–F). This was corroborated by dendritic arbor convex hull area measurements, which showed the dendritic arbors of young and aged mice occupying a similar area (Figure 2, G and H). The dendritic diameter was also unchanged, except for the proximal region, in aged motor neurons (Figure 2I). Thus, aging has a limited effect on the morphology of the dendritic arbor of motor neurons in transgenic Chat-Cre;tdTomato mice.

### The motor circuitry selectively loses excitatory synapses during aging in mice.

**Figure 3:**
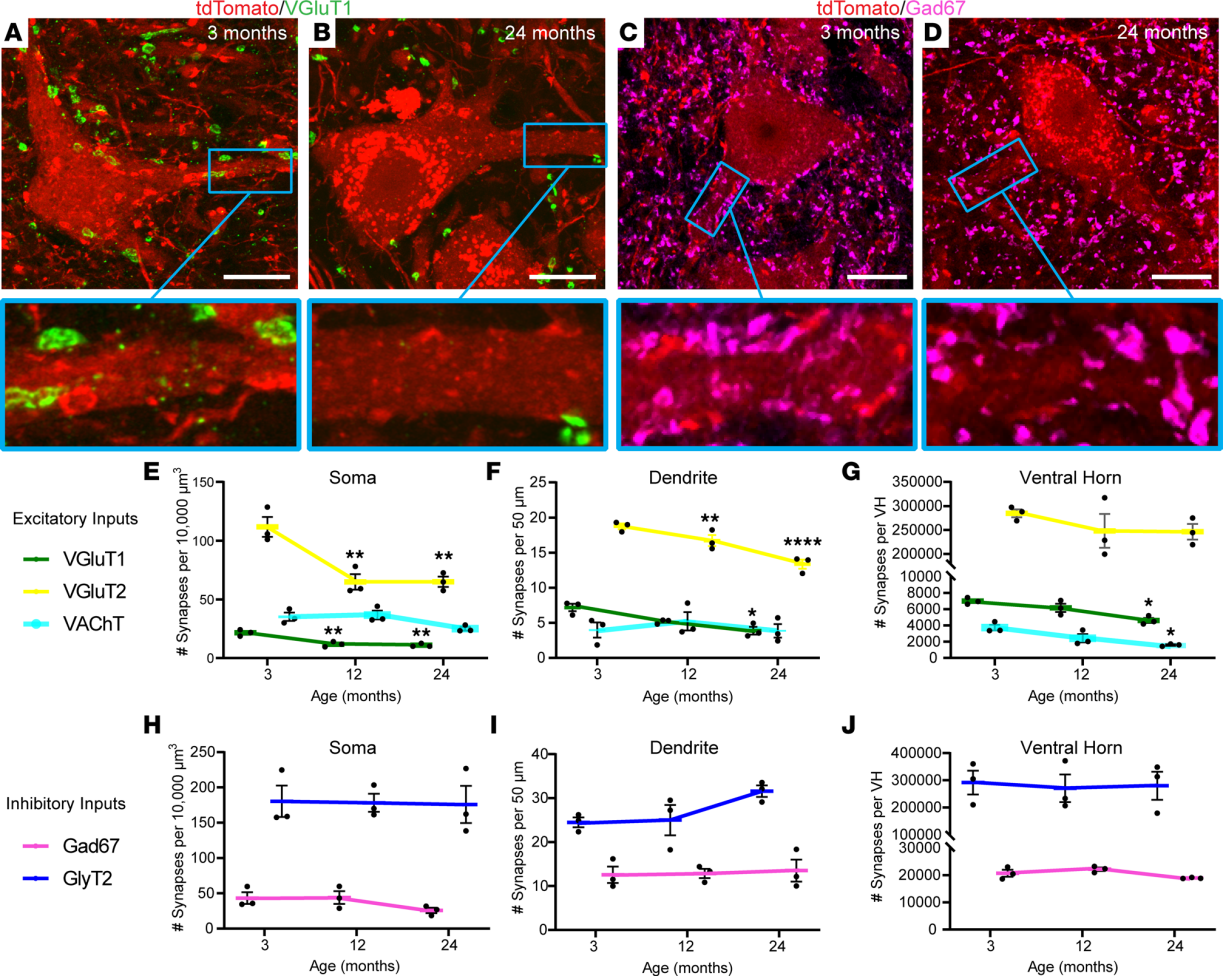
The motor circuitry loses excitatory synapses and remodels during aging. (**A**–**D**) Representative images of VGluT1 (**A** and **B**) and Gad67 (**C** and **D**) puncta on tdTomato-labeled motor neurons in young and aged Chat-Cre;tdTomato mouse lumbar spinal cord. (**E**–**G**) Excitatory VGluT1, VGluT2, and VAChT synaptic density in mouse motor neuron soma (**E**), motor neuron dendrites (**F**), and ventral horn (**G**). (**H**–**J**) Inhibitory Gad67 and GlyT2 synaptic density in mouse motor neuron soma (**H**), motor neuron dendrites (**I**), and ventral horn (**J**). One-way ANOVA with Bonferroni post hoc used for all comparisons. **P < 0.01, ****P < 0.0001 versus 3 months. All values presented as mean ± SEM; *n* = 3. Scale bar: 20 μm.

We asked if the motor circuitry, the compilation of synapses on motor neurons, undergoes significant changes during aging. We examined all major classes of motor neuron synaptic inputs, including cholinergic, glutamatergic, GABAergic, and glycinergic synapses, on the soma and dendritic arbor (Figure 3, A–D, and Supplemental Figure 2, A–F). We used 50 μm–thick L1 lumbar spinal cord sections from young and aged Chat-Cre;tdTomato mice to examine synaptic inputs along the entire surface of the soma and over an average distance of 302 μm of the dendritic arbor (Supplemental Figure 2, G and H). This robust analysis showed that excitatory synapses decrease with increasing age while the number of inhibitory synapses within the motor circuitry remain unchanged. We found decreased numbers of excitatory vesicular glutamate transporter 1^+^ (VGluT1^+^) and VGluT2^+^ synaptic puncta on motor neuron soma (Figure 3E) and dendrites (Figure 3F) as early as 12 months of age. Interestingly, the size of the remaining VGluT1^+^ and VGluT2^+^ synapses is unchanged in aged mice (Supplemental Figure 2, I and J), suggesting that they are suddenly and completely removed. VAChT synaptic inputs trended toward a decreased number on the soma of motor neurons at 24 months of age (Figure 3E), in line with our published observation (24). We also found that VAChT synaptic inputs significantly and progressively decrease in size during aging (Supplemental Figure 2K). Additional analysis showed that the number of these 3 excitatory synaptic inputs decreased significantly or trended lower in the entire ventral horn of aged mice (Figure 3G). By contrast, the number and size of inhibitory glutamic acid decarboxylase 67 (Gad67) and glycine transporter 2 (GlyT2) synaptic inputs on the motor neuron soma (Figure 3H) and dendrites (Figure 3I), as well as throughout the ventral horn (Figure 3J), remain unchanged in aged mice (Supplemental Figure 2, L and M).

### Electron microscopy also reveals fewer synapses on the soma and dendrite of aged motor neurons.

**Figure 4:**
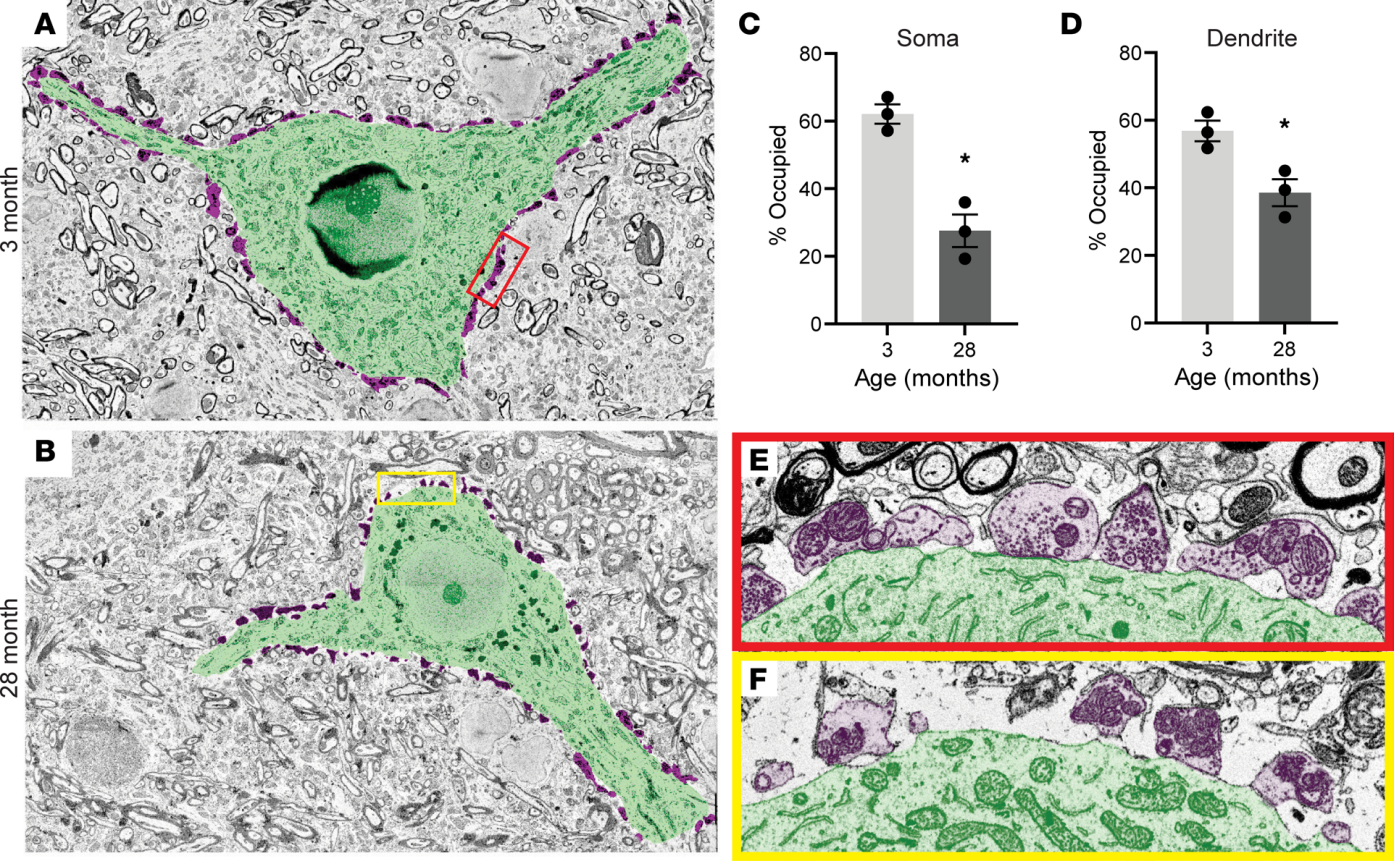
EM analysis of motor neuron synaptic coverage. (**A** and **B**) Representative images of motor neuron soma and proximal dendrites (green) and presynaptic boutons (magenta) in cross section in 3-month (**A**) and 28-month (**B**) mouse lumbar spinal cord. (**C** and **D**) Quantification of soma (**C**) and proximal dendrite (**D**) surface occupancy by synapses. (**E** and **F**) Somatic synapses are surrounded by thin proteoglycan-rich perineuronal network in the 3-month spinal cord. At 28 months, synaptic terminals are fewer and smaller with an overall reduced synaptic occupancy of motor neuron soma. *P < 0.05 versus young, unpaired, 2-sided *t* test. Data points represent individual motor neurons from 1 mouse. All values presented as mean ± SEM; *n* = 3. Scale bars: 10 μm (**A** and **B**) and 1 μm (**E** and **F**).

To validate the light microscopy findings above and generate additional insights, we examined motor neurons and their synapses using serial block face scanning electron microscopy (EM). This allowed us to perform a high-resolution analysis of age-related changes in synaptic inputs on the motor neuron soma and proximal dendrites. At 4 months of age, approximately 60% of the surface of motor neurons was covered by presynaptic boutons (Figure 4, A and C). However, this coverage was reduced to approximately 25% at 28 months (Figure 4, B and C). Likewise, aging reduced the synaptic coverage of proximal dendrites from nearly 60% at 4 months to approximately 40% at 28 months (Figure 4D). The loss of coverage is influenced by an apparent reduction in both the density and the sizes of synaptic boutons (Figure 4, E and F). EM revealed subcellular features at remaining aged synaptic inputs, suggestive of progressive degeneration and dysfunction that include morphological changes and improper apposition with the motor neuron soma (Figure 4, E and F).

### Aging reduces glutamatergic synaptic clustering along the dendritic arbor.

**Figure 5:**
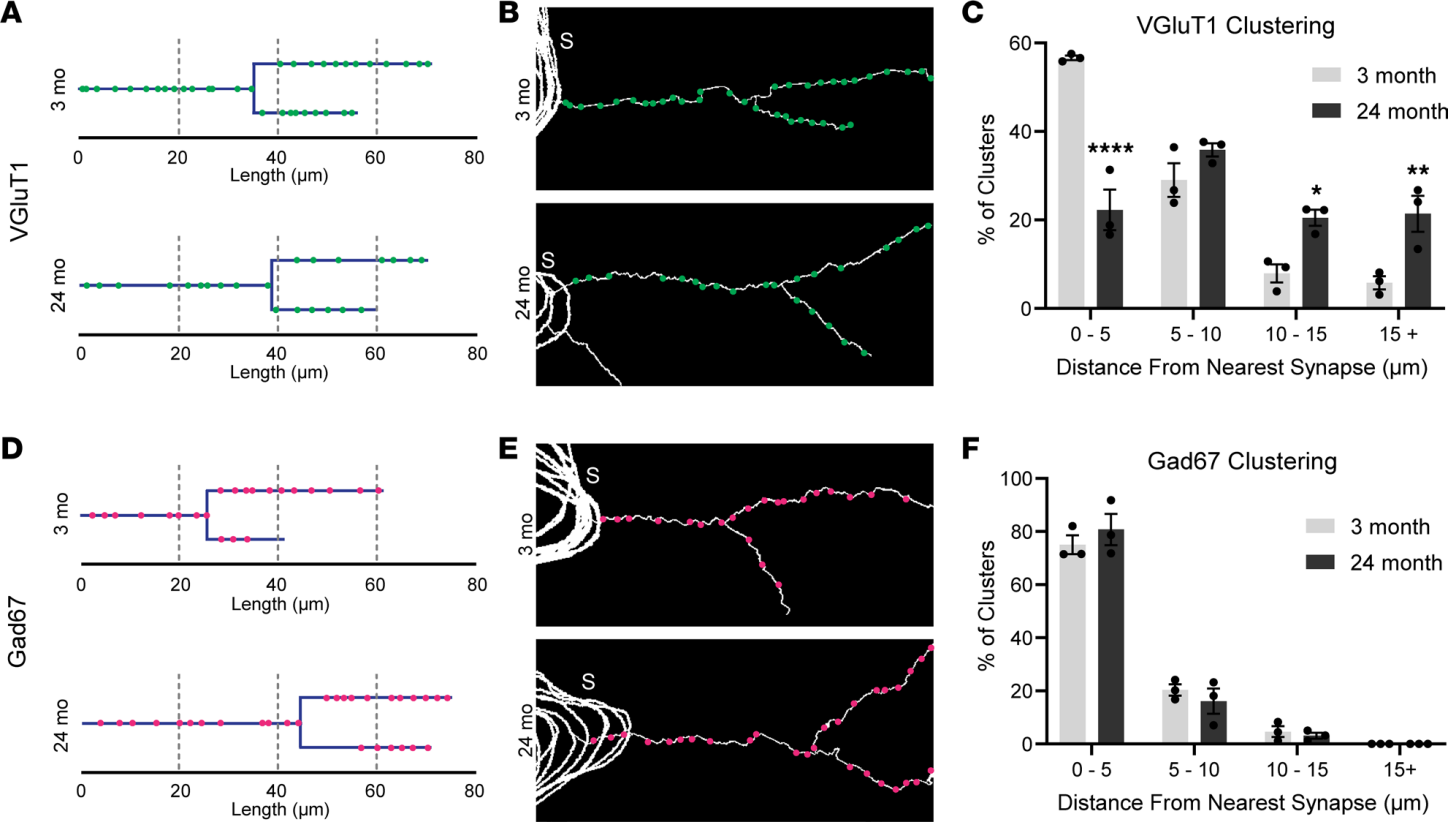
Clustering is reduced in excitatory but not inhibitory inputs on aged motor neurons. (**A**) Schematic representation of the location of VGluT1^+^ glutamatergic synapses along a section of the dendritic arbor of motor neurons from 3- and 24-month-old mice. (**B**) Representative neuronal traces of VGluT1^+^ synapse locations along dendrites. S labels the motor neuron soma. (**C**) Quantification of the percentage of total dendritic synapses that have a nearest neighbor within the specified distance along the occupied dendrite. (**D**) Schematic representation of the location of Gad67^+^ GABAergic synapses along a section of the dendritic arbor of motor neurons from 3- and 24-month-old mice. (**E**) Representative neuronal traces of Gad67^+^ synapse locations along dendrites. S labels the motor neuron soma. (**F**) Quantification of the percentage of total dendritic synapses that have a nearest neighbor within the specified distance along the occupied dendrite. Unpaired, 2-sided *t* test used for all comparisons. *P < 0.05, **P < 0.01, ****P < 0.0001 versus 3 months. All values presented as mean ± SEM; *n* = 3.

The spatial organization of synaptic inputs has been proposed to be important for modulating the function of motor neurons as well as other neurons. Recent findings suggest that alterations to this spatial organization likely occur during periods of synaptic plasticity or neuronal stress. For instance, VGluT1^+^ synaptic input clustering along the dendrites of motor neurons is disrupted following axotomy, with the number of these inputs within 5 μm of their nearest neighbor decreasing significantly in axotomized compared with uninjured motor neurons (42). We thus asked if aging affects the spatial organization of motor synapses in mice. We used the previously defined criteria of quantifying nearest neighbor distances of synapses of the same type and comparing the percentage of dendritic synapses that are within a 5 μm nearest neighbor distance along the dendrite (42). We found VAChT^+^, VGluT1^+^, VGluT2^+^, GlyT2^+^, and Gad67^+^ synapses clustered along the dendrites of young adult motor neurons. In aged motor neurons, we found that the number of closely opposed VGluT1 (Figure 5, A–C) and VGluT2 (Supplemental Figure 3, A–C) synapses decreased. The number of VAChT clusters remained unchanged (Supplemental Figure 3, D–F), while GlyT2 clusters increased (Supplemental Figure 3, G–I) and Gad67 clusters were unchanged (Figure 5, D–F) in aged mice. Thus, aging negatively affected glutamatergic synapses in 2 ways. It decreased the total number as well as their clustering. In contrast, aging increased the number of inhibitory synapse clusters without altering the total number of these synapses.

### In rhesus monkeys and humans, excitatory motor synapses are also lost in old age.

We then asked if motor neurons and the motor circuitry undergo similar changes in aging rhesus monkeys (Supplemental Figure 4, A and B). We examined the density, soma size, and dendrite width of motor neurons in the cervical spinal cords of young (6–11 years) and aged (28 years) rhesus monkeys. We found that there was no age-related change in any of these metrics (Supplemental Figure 4, C–E). We also examined the number and distribution of excitatory VGluT1^+^ and inhibitory Gad67^+^ synaptic inputs along the soma and proximal dendrites of motor neurons. Immunostaining revealed a trend toward a decrease in somatic and dendritic VGluT1^+^ synaptic inputs in aged motor neurons (Supplemental Figure 4F). As in mice, the density of Gad67^+^ synaptic inputs remained unchanged in aged rhesus monkey motor neurons (Supplemental Figure 4H). Analyses of synapses in the ventral horn showed the same trend (Supplemental Figure 4, G and I). These findings support published work from our lab (24) and findings above in mice that only excitatory synaptic inputs decrease with advancing age in both mice and rhesus monkeys.

**Figure 6:**
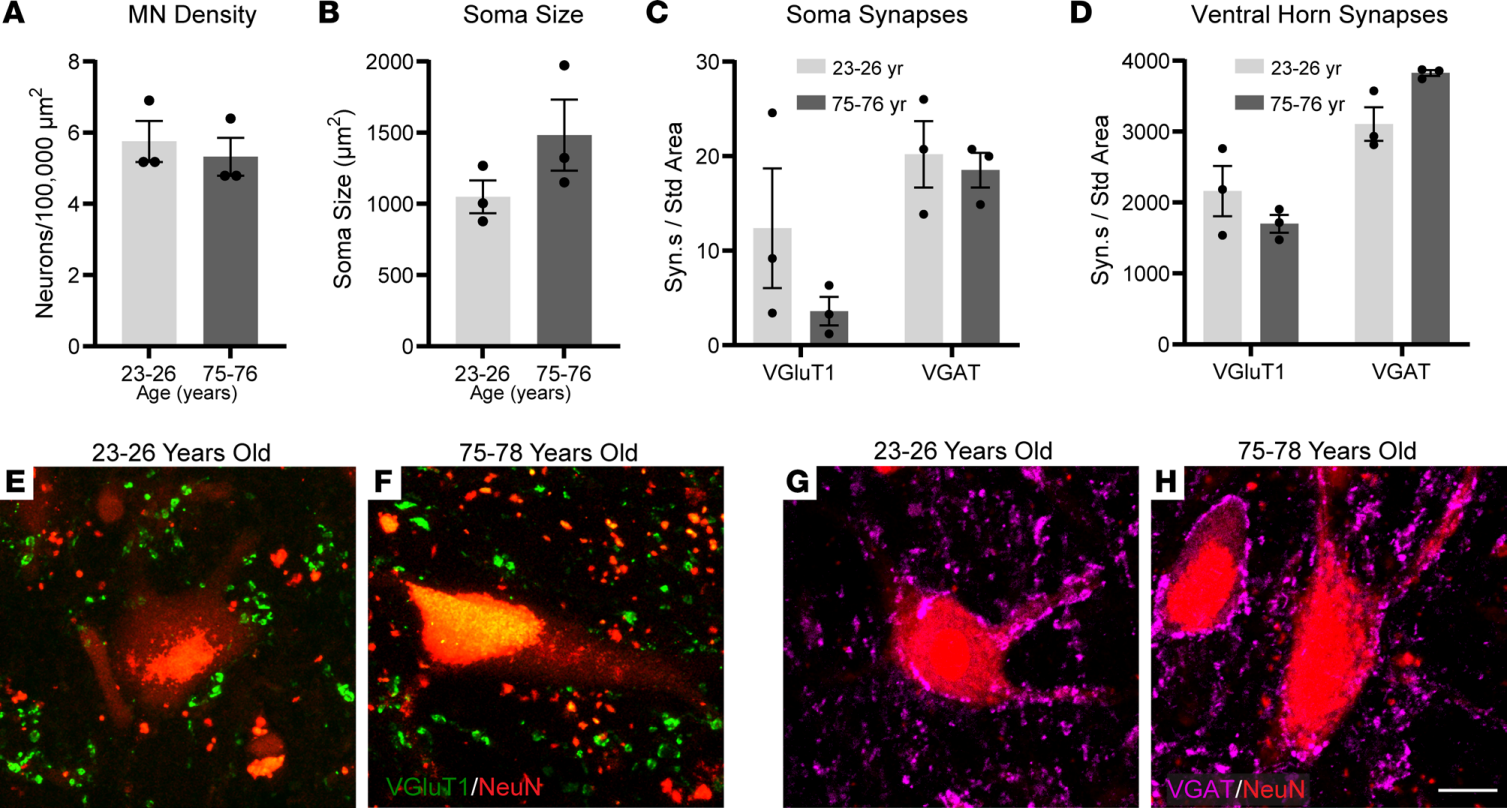
Profiling of human motor neurons and synapses during aging. (**A** and **B**) Quantifications of motor neuron density (**A**) and soma size (**B**) reveal no significant changes with age. (**C** and **D**) The number of VGluT1^+^ and VGAT^+^ synapses on motor neuron soma (**C**) and in the ventral horn (**D**) are unchanged in aged spinal cord; however, a trend toward decreased VGluT1 synapses was observed. (**E** and **F**) Images of motor neurons (NeuN, red) and glutamatergic synapses (VGluT1, green) of young and aged humans. (**G** and **H**) Images of motor neurons (NeuN, red) and GABAergic synapses (VGAT, fuchsia) of young and aged humans. Unpaired, 2-sided *t* test used for all comparisons except **C** (VGluT1) and **D** (VGAT), where an unpaired, 2-sided *t* test with Welch’s correction was used. All values presented as mean ± SEM; *n* = 3. Scale bar: 20 μm.

Importantly, we assessed the impact of aging on motor neurons and their excitatory synaptic inputs in young adult (23–26 years) and elderly (75–76 years) cervical spinal cords of humans. We examined motor neuron density (Figure 6A) and size (Figure 6B) and found no age-related changes. We also examined VGluT1^+^ (Figure 6, C–F) and vesicular GABA transporter (VGAT) inhibitory (Figure 6, C, D, G, and H) synaptic inputs in motor neurons. We observed a trend toward fewer VGluT1^+^ synaptic inputs and no change in VGAT^+^ inputs on the soma of motor neurons (Figure 6C) and throughout the ventral horn (Figure 6D) of aged human spinal cords. Overall, these analyses in mice, rhesus monkeys, and humans show that the number of excitatory synaptic inputs on motor neurons decreases while inhibitory synaptic inputs remain unchanged during aging.

### Identification of molecular features of aging motor neurons.

**Figure 7:**
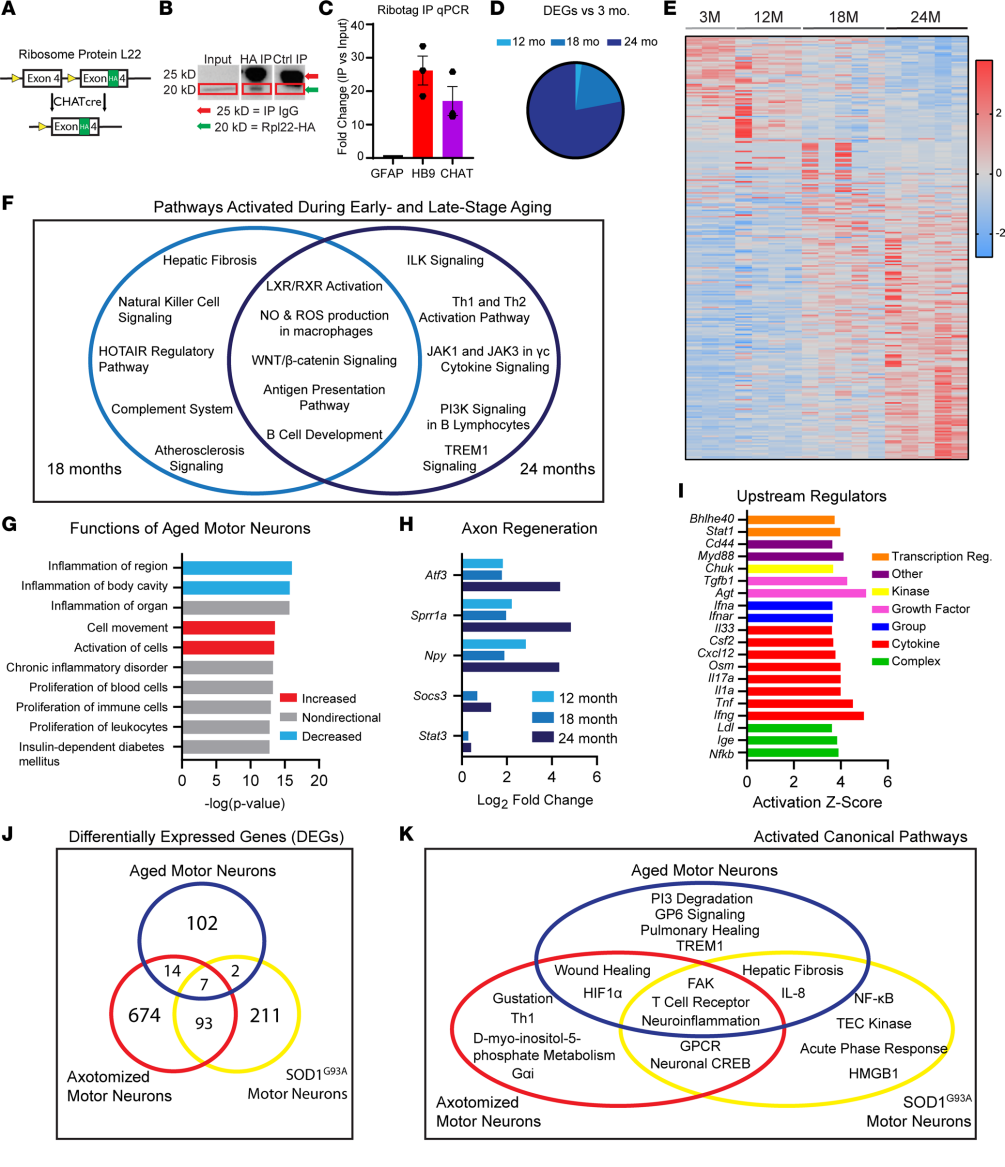
Molecular profile of aged motor neurons. (**A**) The modified exon 4 sequence containing an HA tag is incorporated into the RPL22 gene following Cre expression under the *Chat* promoter in Chat-Cre;RiboTag mice. The endogenous exon 4 contains a stop codon. (**B**) Western blot analysis of whole-spinal cord input tissue and immunoprecipitated ribosomes reveals enrichment of Rpl22-HA by IP. Lanes were run on the same gel but were noncontiguous. (**C**) qPCR analysis of *Gfap*, *Hb9*, and *Chat* transcripts in RNA co-IP versus input fractions. (**D**) Proportion of differentially expressed genes (DEGs), relative to 3-month young adult controls, among 12-, 18-, and 24-month motor neuron transcriptomes. (**E**) Heatmap of read count *Z* scores of DEGs. (**F**) Ingenuity pathway analysis (IPA) of canonical pathways activated in early-stage (18 month) and late-stage (24 month) aged motor neurons. (**G**) Top IPA functions of 24-month motor neurons. (**H**) Expression levels of regeneration-associated genes in 12-, 18-, and 24-month motor neurons, as compared with 3 months of age. (**I**) Top upstream regulators of 24-month motor neurons. (**J**) Comparison of common DEGs in aged, axotomized, and 4-month-old SOD1^G93A^ motor neurons. (**K**) Shared IPA canonical pathways in aged, axotomized, and 4-month-old SOD1^G93A^ motor neurons. Values in **C** are presented as mean ± SEM. Values in **G**–**I** are presented as mean; *n* = 3–5.

The molecular profile of aged spinal motor neurons has not been defined, to date. This information may be critical to prevent synaptic loss and other age-related changes in the motor system. To interrogate the molecular composition of aging motor neurons, we used RiboTag mice to specifically isolate midtranslation mRNA from motor neurons. RiboTag mice conditionally express hemagglutinin-tagged ribosomal protein L22 (HA-Rpl22) (43) (Figure 7A). We crossed this line with Chat-Cre mice to generate Chat-Cre;RiboTag mice. These mice were used to immunoprecipitate HA-Rpl22 ribosomes from Chat^+^ cholinergic neurons, of which motor neurons comprise a significant proportion, in the spinal cord. We confirmed presence of HA-Rpl22 protein in the spinal cord of Chat-Cre;RiboTag mice with an anti-HA immunoblot of immunoprecipitated ribosomes (Figure 7B; see complete unedited blots in the supplemental material). Importantly, we validated that isolated HA-Rpl22 ribosomes contain high levels of the motor neuron–specific transcripts Homeobox HB9 (*Hb9*) (44) and *Chat* (45) but lack transcripts for glial fibrillary acidic protein (*Gfap*), an astrocytic marker (46) (Figure 7C).

We then profiled the translatome — all the mRNAs associated with ribosomes and, hence, in the process of being translated — of aging motor neurons from male and female Chat-Cre;RiboTag mice using RNA-Seq. We identified differentially expressed genes (DEGs) in aging motor neurons using a minimum read count of 5 and a *P* value of less than 0.05. In males, this analysis revealed a progressive age-related shift in the motor neuron translatome with the greatest proportion of DEGs found in 24-month-old compared with 3-month-old motor neurons (Figure 7, D and E). Upregulated genes comprised the majority of DEGs in 24-month-old motor neurons (133 of 142), as compared with motor neurons 3 months of age (Supplemental Figure 5). Among the downregulated DEGs in this age group (Supplemental Table 1) is *Htr1b*, which encodes the 1B serotonin receptor. Deletion of this gene in mice accelerates age-related loss of motor function (47). Other downregulated genes include *Ppp1r1c*, which has recently been shown to be a marker of α-motor neurons (48), and *Sfrp1*, which encodes a secreted protein that modulates microglial activation (49).

Ingenuity Pathway Analysis (IPA) of DEGs in 18- and 24-month-old motor neurons, as compared with motor neurons 3 months of age, revealed signaling pathways with roles in synaptic reorganization and plasticity (Figure 7F) — including IL-linked kinase (ILK) (50), WNT/β-catenin (51, 52), and complement (53, 54) — increased in the aged motor neuron translatome. IPA also suggests that aged motor neurons reduce expression of inflammatory genes but become prone to interactions with immune cells and to activating cell movement pathways, possibly in an attempt to regenerate their axonal terminals at NMJs (22) (Figure 7G). Among DEGs in aging motor neurons, a number have been previously shown to promote motor neuron axonal regeneration following axotomy or in ALS mouse models. These genes include *Atf3* (55), *Sprr1a* (56), and *Npy* (57) (Figure 7H). Additionally, IPA shows that predicted upstream regulators of the aging motor neuron translatome include cytokines, such as *Il33*, *Tnf*, and *Cxcl12*, the transcriptional regulators *Stat1* and *Nfkb*, and *Tgfb* (Figure 7I). Furthermore, we compared DEGs in 24- versus 3-month-old motor neurons with published data sets of transcriptional changes in axotomized and ALS-afflicted motor neurons (58). We found DEGs that are unique to each group, including 102 genes uniquely upregulated in aged motor neurons. We also found a number of genes that are shared between conditions and found 7 shared among all 3 conditions (Figure 7J), including *C1qa*, *Cd63*, *Npy*, *Serpinb1a*, *Sprr1a*, *Tyrobp*, and *Vim*. Many of the genes shared with one or both of these conditions are common neuronal stress-related genes, such as *Atf3* (59). We also utilized these data sets to compare canonical pathways that are unique and shared between conditions. This comparison revealed that GP6 signaling, a pathway implicated in schizophrenia (60), and TREM1 signaling, which is related to inflammation and glial activation (61), are uniquely activated in aged motor neurons (Figure 7K).

**Figure 8:**
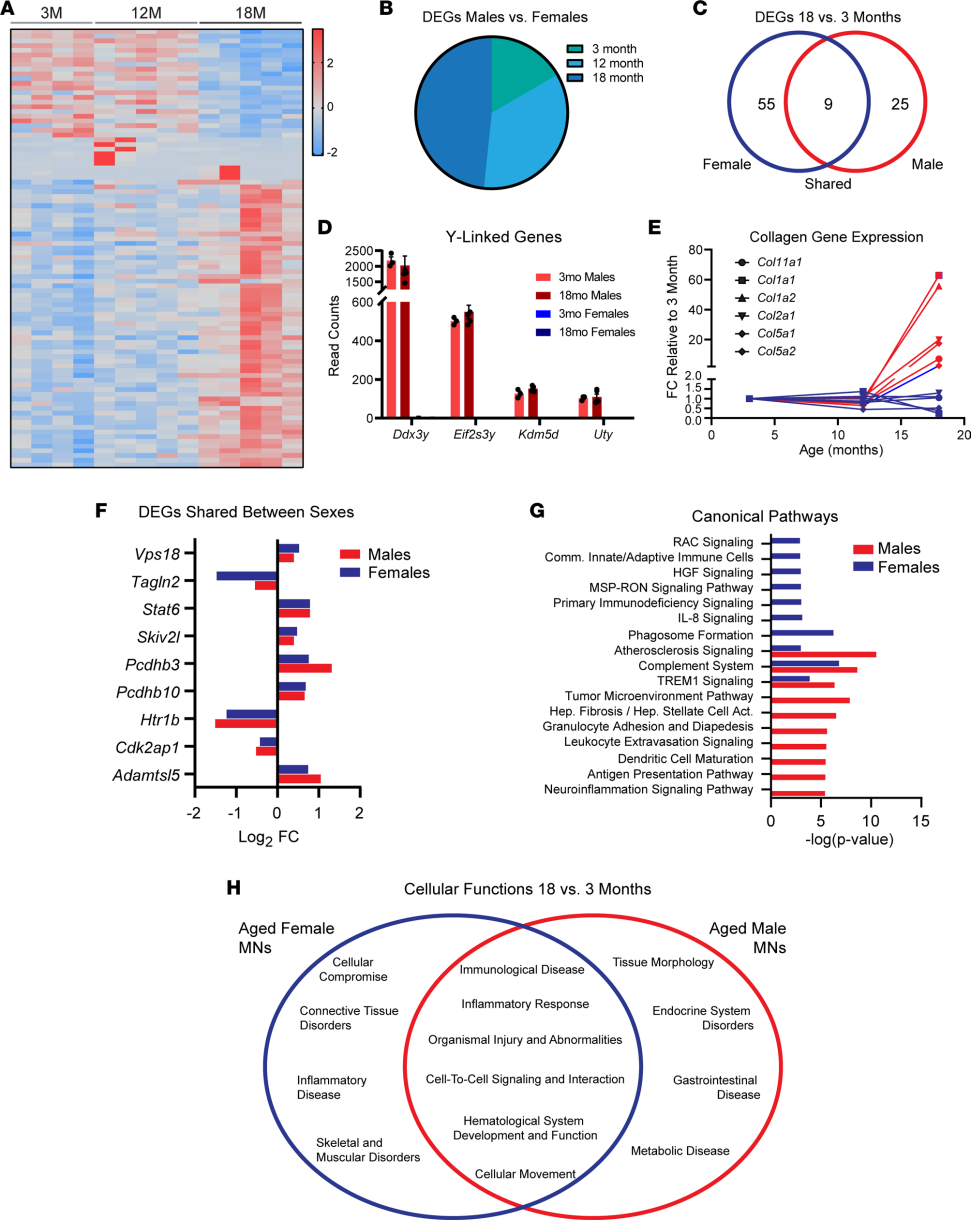
Molecular profile of aged male and female motor neurons. (**A**) Heatmap of read count *Z* scores of DEGs in female motor neurons. (**B**) Numbers of DEGs in males versus female motor neurons at 3, 12, and 18 months of age. (**C**) Comparison of the number of common and unique DEGs. (**D**) Read counts of Y-linked genes in young and old male and female motor neurons. (**E**) Collagen genes are upregulated only in male motor neurons at 18 months old. (**F**) Fold change of shared DEGs between sexes shows that the magnitude of change is similar. (**G**) Shared and unique activated canonical pathways identified via IPA analysis in male and female motor neurons. (**H**) Shared and unique activated functions identified via IPA analysis in male and female motor neurons. Values in **D** are presented as mean ± SEM. Values in **E**, **F**, and **H** are presented as mean; *n* = 3–5.

To account for sex differences, we probed the translatome of female motor neurons in 3-, 12-, and 18-month-old Chat-Cre;RiboTag mice. Not surprisingly, the female motor neuron translatome was also altered by aging (Figure 8A and Supplemental Figure 6). We then compared the female data set with that from male mice up to 18 months of age. We found that there are increasing numbers of DEGs between male and female motor neurons at older ages (Figure 8, B and C). As expected, Y-linked genes were exclusively expressed in males (Figure 8D). We also found several collagens enriched in males compared with females at 18 months of age (Figure 8E). However, several age-related DEGs were shared by both sexes, in both their magnitude and directionality (Figure 8F). IPA analysis showed that many of the DEGs in aged female motor neurons are linked to canonical pathways (Figure 8G) and cellular functions (Figure 8H) unique to females and are also shared with males. Of potential relevance to age-related loss of motor synapses, the translatomes of both aged female and male motor neurons are enriched for genes with roles in the complement system and TREM1 signaling (Figure 8H). Both complement signaling (62–66) and TREM1 (67, 68) have been shown to mediate synaptic pruning by microglia; thus, motor neurons undergo molecular changes during aging that may cause microglia to target motor synapses.

To validate RNA-Seq findings, we examined expression of *Sprr1a* (Figure 7H) in young and aged mouse motor neurons using RNAscope (Supplemental Figure 7, A and B). This method showed that *Sprr1a* is highly expressed in a subset of aged motor neurons and absent from young adult motor neurons (Supplemental Figure 7C). We also examined expression of apolipoprotein E (*Apoe*), one of the most highly expressed and upregulated genes in motor neurons, in 24-month-old mice. We first used IHC to determine whether motor neurons in fact express and increase levels of ApoE protein during aging (Supplemental Figure 7, D and E). This analysis found ApoE trending toward elevated levels in aged motor neurons (Supplemental Figure 7F). We then used quantitative PCR (qPCR) to analyze *Apoe* levels in immunoprecipitated ribosomes from motor neurons of young and aged Chat-Cre;RiboTag mice. *Apoe* was again found elevated in aged motor neurons (Supplemental Figure 7G). These *Apoe* and *Sprr1a* findings strongly suggest that motor neurons age in a heterogeneous manner or at different rates within the same animal and spinal cord region.

## Discussion

### Summary and potential implications.

This is the first study to our knowledge to holistically examine, on a cellular and molecular level, the impact of aging on motor neurons within the spinal cord of mice, rhesus monkeys, and humans. We used complementary techniques, including PACT-CLARITY tissue clearing, to reveal the cellular features of the soma and dendritic arbor of aged motor neurons. We demonstrate that spinal motor neurons were surprisingly resilient to aging in mice, monkeys, and humans. The number and overall morphology of these neurons did not significantly change with advancing age. However, these neurons progressively and selectively shed excitatory synaptic inputs from the soma and dendritic arbor. Thus, aged motor neurons contain a motor circuitry with a reduced ratio of excitatory to inhibitory synapses, which may be responsible for the diminished ability to activate motor neurons to commence movements. We also examined the motor neuron translatome (ribosomal transcripts) in both male and female mice. This strategy identified genes and molecular pathways with roles in glia-mediated synaptic pruning, axonal regeneration, inflammation, and oxidative stress. Some of these genes and pathways are also found altered in motor neurons affected with ALS or responding to axotomy, demonstrating that aged motor neurons are under substantial stress. Additionally, it revealed molecular similarities and differences between aging male and female motor neurons across the lifespan. Thus, this approach identified molecular pathways intrinsic to motor neurons that may contribute to degeneration of the motor circuitry and the NMJ during aging.

### Likely source of motor dysfunction in the aged spinal cord.

The literature concerning whether motor neurons are lost or atrophy during aging has been inconsistent for decades (17, 19–23, 26–33). While published studies have attempted to quantify motor neuron number and size, many did so via indirect or nonspecific labeling techniques and with small sample sizes. In this study, we sought to contribute new evidence to this debate by using several methods that directly label motor neurons. Each of our approaches revealed that there was no difference in the number of motor neurons between young and aged spinal cords across species. However, it is worth noting that aging caused a significant reduction in the size of motor neurons in Chat-Cre;tdTomato but not Wt mice. A frequency distribution analysis indicated that the largest motor neurons are preferentially affected and, therefore, account for the reduced size in aged Chat-Cre;tdTomato mice. The most parsimonious explanation for this observation is that expression of tdTomato may increase the susceptibility of large motor neurons to the stress of aging. This possibility is supported by a study showing that motor neurons expressing another fluorescence protein, YFP, prematurely exhibit signs of aging along their axons (69). The largest motor neurons are also most sensitive to ALS-induced stress, further lending support to the possibility that expression of tdTomato is a stressor to motor neurons, although minor since only their size was affected. Altogether, however, our data show that aging does not significantly alter the number of motor neurons. Aging also does not cause major changes to the morphology of motor neurons’ dendritic arbors. We therefore conclude that other age-related changes within the motor circuitry impair the capacity to commence and modulate movements in aged animals and humans.

Our data suggest that loss of excitatory synapses may be responsible for the compromised function of aged motor neurons in animals and humans. We found significant decreases in the number and clustering of both VGluT1^+^ and VGluT2 ^+^ synaptic inputs innervating aged motor neurons. We also found VAChT^+^ synaptic inputs trending lower in aged motor neurons. These findings are consistent with previous published studies from our group (24) and other groups (70), showing fewer excitatory synapses at the soma of aged motor neurons. Loss of excitatory synaptic inputs and clusters is also a feature of axotomized motor neurons (42), which is not surprising, given that aging also leads to degeneration of motor axon endings at NMJs (4). Overall, these findings show that aged motor neurons shed excitatory synapses, likely compromising the fidelity of motor commands emanating from proprioceptive sensory, cortical, subcortical, and spinal cord neurons.

In stark contrast to excitatory synaptic inputs, we found the number and clustering of inhibitory synaptic inputs either unchanged or rather moderately increased at aged motor neurons. Thus, aged motor neurons have a higher ratio of inhibitory to excitatory synapses, suggesting that they may have an increased threshold and latency for activation. This would, in turn, reduce the number of motor neurons recruited to relay motor commands. The higher threshold to activate motor neurons due to the increased proportion of inhibitory synapses, along with selective degeneration of some motor axon nerve endings at NMJs (14), may be a reason fewer motor units are found in old age (71). These changes would, in turn, reduce the number of muscle fibers recruited to perform a particular motor task. It may also delay the initiation and coordination of movements, resulting in poor balance and gait abnormalities. Support for these possibilities can be found in other published studies showing that motor deficits begin to be evident by middle age in mice and humans (72–74). However, it is possible that motor neurons alter their biophysical properties to adapt to changes in the motor circuitry to continue to integrate and relay motor commands without delays. Even if motor neurons undergo homeostatic adaptations to counteract the loss of excitatory synapses, they will fail to receive critical motor commands from many proprioceptive sensory neurons as well as excitatory cortical, subcortical, and spinal cord neurons. Thus, the loss of excitatory synaptic inputs alone likely compromises the fidelity of motor commands and, thus, would contribute to motor deficits, during aging.

### Molecular mechanisms underlying age-related changes in motor circuits.

Our translatome analysis identified specific genes and pathways, some with known roles at synapses, predicted to be activated or deactivated in aging motor neurons. It showed that complement signaling was activated in aged motor neurons, which has been well described as a microglia-mediated synaptic pruning mechanism (54, 75–77). Thus, motor neurons may cause the loss of their own synapses during aging by aberrantly activating microglia through complement signaling. We also found that the WNT/β-catenin signaling pathway was activated during both early- and late-stage aging. This pathway has been highly implicated in mediating synaptic plasticity (78), and reactivation of this pathway has been touted as a potential therapeutic intervention in Alzheimer’s disease (79). Interestingly, the predicted outcome of WNT/β-catenin signaling is increased intrinsic excitability (80). Thus, aged motor neurons may recruit this signaling pathway in response to the increased inhibitory tone to restore normal levels of excitability. Additionally, aged motor neurons may activate WNT/β-catenin signaling as well as the PI3K and ILK pathways, which have been suggested to act in conjunction to influence structural plasticity in the aged brain (50) in order to rewire the motor circuitry in response to loss of excitatory synaptic inputs.

The translatome data also indicate that a “tipping point” may not exist for motor neurons as they transition from a youthful to an old state. Rather, the translatome of motor neurons continuously and gradually changes throughout aging. While these changes may be continuous; they are not homogenous in nature. There are genes and pathways differentially expressed between middle-aged and old motor neurons, indicating that motor neurons deal with challenges unique to a given stage of the aging process by modulating specific genes and pathways.

We also report, for the first time to our knowledge, molecular differences and similarities between male and female motor neurons. Of particular note is our finding that collagen genes are significantly upregulated in aged male motor neurons but not in females. This suggests that male motor neurons may experience peripheral axonal degeneration first and, thus, activate genes intended to rebuild the extracellular matrix as the motor neurons attempt to regenerate and reinnervate their muscle targets. Altogether, these molecular and cellular discoveries demonstrate how aging affects motor neurons and provide leads to preserve voluntary movements during normal aging.

## Methods

### Mice

RiboTag (RRID:IMSR_JAX:011029; ref. 43), Chat-Cre (RRID:IMSR_JAX:006410; ref. 81), and Ai14 (herein referred to as tdTomato, RRID:IMSR_JAX:007914; ref. 82) mice were purchased from the Jackson Laboratory. C57BL/6 mice were obtained from the National Institute on Aging at the NIH. Hemizygous Chat-Cre mice were crossed with tdTomato mice to generate Chat-Cre;tdTomato mice, and with RiboTag mice to generate Chat-Cre;RiboTag mice. All mice were maintained on a mixed genetic background. Male and female mice were included in experiments, as described in the results. A CO_2_ chamber was used to sacrifice mice for tissue collection, following NIH guidelines. Animals were housed in a 12-hour light/dark cycle with access to food and water ad libitum in a pathogen-free environment.

### Human spinal cord tissue

Fixed postmortem cervical spinal cord tissue from young and aged subjects with a clinical brain diagnosis of unaffected control was obtained from the University of Pittsburgh’s NIH NeuroBioBank Brain and Tissue Repository. Details of subjects are provided in Table 1. Coronal sections (50 μm) were collected with a vibratome and stored in PBS. Sections were washed 3***×*** in PBS, incubated in blocking buffer (5% BSA, 5% donkey serum, 0.05% Triton X-100 in PBS) for 1 hour at room temperature, incubated in primary antibody diluted in blocking buffer 48 hours at 4°C, washed 3***×*** in PBS, incubated in secondary antibody diluted 1:1,000 in blocking buffer for 4 hours at 4°C, washed 3***×*** in PBS, mounted on gelatin covered slides, and covered in VECTASHIELD mounting medium (Vector Laboratories). Primary antibodies include mouse anti-NeuN (1:500, MilliporeSigma, MAB377, RRID: AB_2298772) and rabbit anti-vGlut1 (1:500, Synaptic Systems, 135303, RRID: AB_887875). Secondary antibodies include Alexa Fluor 488 goat anti-mouse (Invitrogen, A21121, RRID: AB_2535764) and Alexa Fluor 568 goat anti-rabbit (Thermo Fisher Scientific, A11011, RRID: AB_143157).

### Nonhuman primate spinal cord tissue

Rhesus monkey (Macaca mulatta) spinal cord samples were obtained from the National Institutes of Health Animal Center. Samples were collected from males following euthanasia for unrelated clinical or experimental purposes; collection occurred within 2 hours of death. Once harvested, samples were immersed in ice-cold 4% paraformaldehyde (PFA) for 24–48 hours and then kept in cold 0.1 M PBS. Sectioning and IHC of nonhuman primate tissue was performed identically to C57BL/6 mouse spinal cord tissue, as described below. Antibodies include mouse anti-Gad67 (1:500, MilliporeSigma, MAB5406, RRID: AB_2278725), rabbit anti-VGluT1 (1:500, Synaptic Systems, 135303, RRID: AB_887875), and rabbit anti-VGAT (MilliporeSigma, AB5062P, RRID: AB_2301998).

### Mouse tissue

Mice were intracardially perfused with 1***×*** PBS, followed by 4% PFA. Spinal cords were dissected and postfixed for 4 hours at 4°C. Tissue clearing was performed on spinal cords collected from Chat-Cre;tdTomato mice according to Woo et al (41). Briefly, 500 μm coronal sections from the L1–L4 lumbar regions of the spinal cords were collected with a vibratome for clearing. Spinal cord sections were then embedded in a hydrogel and underwent several washes to elute lipids from the scaffold, rendering the samples translucent and significantly reducing refraction. Spinal cords collected from C57BL/6 mice and a separate cohort of Chat-Cre;tdTomato mice that underwent all IHC assays were not subjected to tissue clearing. The L1–L6 regions of the lumbar spinal cord from these mice were embedded in Tissue Freezing Medium (General Data, TFM-5) and 30 μm coronal sections were obtained with a cryostat. All spinal cord sections were mounted to gelatin coated slides, washed 3***×*** in PBS, incubated in blocking buffer (5% BSA, 3% goat serum, and 0.1% Triton X-100 in PBS); donkey serum was substituted for goat serum when necessary) for 1 hour at room temperature, incubated in primary antibody diluted in blocking buffer overnight at 4°C, washed 3***×*** in PBS, incubated in secondary antibody diluted 1:1,000 in blocking buffer for 1 hour at room temperature, washed 3***×*** in PBS, and covered in VECTASHIELD mounting medium (Vector Laboratories). Primary antibodies include goat anti-ChAT (1:500, MilliporeSigma, AB144P, RRID: AB_2079751), mouse anti-Gad67 (1:500, MilliporeSigma, MAB5406, RRID: AB_2278725), rabbit anti-Glyt2 (1:2,500, Synaptic Systems, 272003, RRID: AB_2619997), mouse anti-NeuN (1:500, MilliporeSigma, MAB377, RRID: AB_2298772), guinea pig anti-VAChT (1:500, MilliporeSigma, AB1588, RRID: AB_11214110), rabbit anti-vGlut1 (1:500, Synaptic Systems, 135303, RRID: AB_887875), guinea pig anti-vGlut2 (1:500, MilliporeSigma, AB2251-I, RRID: AB_2665454), guinea pig anti-GFAP (1:500, Nittobo Medical, FR101860, RRID: AB_2571709), and goat anti-ApoE (1:2,500, MilliporeSigma, AB947, RRID: AB_2258475). Secondary antibodies include Alexa Fluor 488 AffiniPure donkey anti–guinea pig IgG (H+L), (1:1,000, Jackson ImmunoResearch, 706-545-148, RRID: AB_2340472), Alexa Fluor 488 goat anti–mouse IgG2a (1:1,000, Invitrogen, A21131, RRID: AB_2535771), Alexa Fluor 647 goat anti–mouse IgG1 (1:1,000, Invitrogen, A21240, RRID: AB_2535809), and Alexa Fluor 488 donkey anti–rabbit IgG (H+L), (1:1,000, Invitrogen, A21206, RRID: AB_2535792).

### Confocal microscopy

Images were obtained with a Zeiss LSM 900 laser scanning confocal microscope (Carl Zeiss Microscopy) using a 63***×*** (1.4 numerical aperture) or 20× (0.8 numerical aperture) objective. Maximum intensity projections and stitching of tile scans were generated with Zeiss Zen software (Carl Zeiss Microscopy, RRID:SCR_013672).

### Serial blockface EM

Sample preparation as well as imaging and data extraction were undertaken at the Cleveland Clinic (Cleveland, Ohio, USA) 3D-EM Ultrastructural Imaging and Computation Core. Unless otherwise noted, all reagents and materials were purchased from Electron Microscopy Sciences (EMS).

#### Sample preparation.

Serial blockface imaging samples were prepared using an ASP1000 robotic stainer (Microscopy Innovations). Samples were inserted into the staining capsules. Using a programmed sequence (available from Microscopy Innovations), they were washed in 0.1 M sodium cacodylate, incubated in a mix of 2% osmium tetroxide with 0.15% potassium ferrocyanide in 0.1 M sodium cacodylate buffer for 2 hours on ice, washed in H_2_O, and then incubated in 0.1% thiocarbohydrazide at 60°C for 45 minutes. They were washed and incubated in 2% osmium tetroxide for at least 2 hours. They were washed again and incubated for at least 48 hours in aqueous Uranyl Acetate (saturated) at 4°C. After washes, they were incubated in lead aspartate stain, dehydrated in graded ethanols, washed in propylene oxide, and then incubated for 4–16 hours in 50:50 resin/solvent. They were then embedded in Epon 812–equivalent resin (Embed 812, Electron Microscopy Services) and cured for 48 hours. Samples were trimmed under a dissecting scope and imaged.

#### Sample imaging.

Samples were imaged in 2 instruments. High-resolution montaged sets were generated in a Thermo Fisher Scientific (FEI) Volumescope2 system. Images were acquired at 2.0 kV in high-vacuum mode using T1 detector and Volumescope stage. Fields were imaged at 5 nm/pixel magnification, and images of 3,000 ***×*** 5,000 pixels wide were stitched to produce slices of 170 μm. Sets of 300–600 slices were collected at cut thicknesses of 50–60 nm, using MAPS2 software (Thermo Fisher Scientific). Montaging sets of small-image tiles reduced sample/beam drift. Samples were also imaged with a Zeiss Sigma VP scanning electron microscope (Carl Zeiss Microscopy) with a 3View in chamber ultramicrotome (Gatan/Amaritech). These were imaged at 2.0 kV, using a 30 μm aperture in high-vacuum mode. Large fields of 10,000 ***×*** 12,000 pixels covered an area of 130 μm wide and did not require stitching. A series of 300–500 slices was generated with 60 nm slices and 7 nm/pixel resolution. The high-resolution sets were used to follow fine structures, while lower-resolution sets provided more rapid analysis of synaptic bouton occupancy of neuronal somas.

#### Analysis.

Image stacks were aligned, cropped, and scaled as necessary using ImageJ/Fiji (NIH) software. Images were examined, annotated and measured using Reconstruct software (83). Three neurons from each spinal cord were assessed in a transverse section through the center of the cell and in slices through proximal dendrites in 4-month and 28-month spinal cords. For each motor neuron, a transverse section through the central neuronal soma was selected, and the synapses making contact with that slice were traced; the proportion of the neuronal surface that they occupied was also annotated. In this context, “occupied” included active zones of synapses and any other part of the axon that was in close apposition to the surface. To illustrate the surface occupancy, different images containing dendrites and cell somas were combined digitally using GNU image processing (GIMP) software.

### Mouse motor neuron morphology

Analysis of motor neurons was performed on stitched, tile scan, *Z* stack confocal images of lumbar spinal cord coronal sections. Motor neuron density and soma size analysis was performed on spinal cord sections following NeuN/VAChT IHC, ChT IHC, or *Chat* RNAScope (density only) in WT mice or in cleared Chat-Cre;tdTomato mice. The number of sections analyzed for each method were as follows: NeuN/VAChT IHC (2 sections, beginning at L1), ChT IHC (2 sections, beginning at L1), *Chat* RNAScope (2 sections, beginning at L1), or Chat-Cre;tdTomato (8 sequential 500 μm sections, beginning at L1). In cleared Chat-Cre;tdTomato spinal cord, significant loss of tdTomato signal was typically observed at 300 μm below the tissue surface. Therefore, tissue at a depth of 300–500 μm was excluded from the analysis. In addition to labeling methods, motor neurons were identified by morphology, soma size, and location within the ventral horn. A grid was applied to the image using ImageJ for random selection of motor neurons for soma size analysis. Average numbers of motor neurons per ventral horn analyzed for soma size were as follows: NeuN/VAChT IHC (10.5 motor neurons), CHT IHC (6.8 motor neurons), *Chat* RNAScope (9.3 motor neurons), Chat-Cre;tdTomato (551.6 motor neurons). All neurons within each imaged section were included for motor density analysis. Soma volume and dendritic arbor complexity analyses were performed on a single cleared 500 μm coronal section from Chat-Cre;tdTomato mice. To calculate soma volume, the soma area of a given motor neuron was measured in serial optical sections along the entire *z* axis of the soma using ImageJ. Each optical section area was multiplied by the optical section height to obtain the soma volume within a given optical section. The volumes of the entire optical section series were then summed to estimate the total soma volume. To calculate dendritic arbor complexity, all visible dendrites of a given motor neuron were traced using the nTracer ImageJ plugin (84). Sholl analysis and convex hull area measurements of dendritic arbors were performed on traces using ImageJ. The diameters of all visible dendrites of a given motor neuron were measured at linear distances of 25, 100, and 150 μm from the soma using ImageJ.

### Synaptic density and clustering

Analysis of synaptic density was performed on images acquired from 50 μm–thick coronal spinal cord sections following Gad67, GlyT2, vGluT1, or vGluT2 IHC. Spinal cord sections were collected via cryostat sectioning and imaged in Chat-Cre;tdTomato mice as described above. Three α-motor neurons per animal were randomly selected using the grid function in ImageJ for analysis. To calculate dendritic synaptic density, the entire visible length of all dendrites of a given motor neuron were traced, and the positions of all synaptic puncta along the traced dendrites were marked using the nTracer ImageJ plugin (84). The total number of synaptic puncta was divided by the total length of dendrites to calculate dendritic synaptic density. To calculate somatic synaptic density, the soma was traced in serial optical sections using ImageJ, and the volume was calculated as described above. Synaptic puncta located at the soma perimeter within each optical section were counted and summed for the entire optical section series. Puncta that spanned more than one optical section were only counted once. Somatic synaptic density was calculated by dividing the total number of puncta by the soma volume. Synaptic puncta in the ventral horn were counted using the analyze particles tool in ImageJ and standardized by the visible area of the ventral horn in each image.

### Analysis of human and monkey tissue

Analyses of human and monkey synaptic density and motor neuron morphology were performed on coronal spinal cord sections following NeuN IHC using the methods described above. NeuN-labeled motor neurons were identified by morphology, soma size, and location within the ventral horn. Cervical monkey spinal cords were fixed and cryosectioned at 50 μm thickness for IHC. Two sections were analyzed for motor neuron density, with an average of 15.6 neurons analyzed per animal. On average, 3 motor neurons were analyzed for soma size, dendrite width, and synaptic density. Cervical human spinal cords were vibratome sectioned at 50 μm thickness, and IHC was performed while sections were free-floating in 12-well plates. Two sections were analyzed for motor neuron density, with an average of 15.1 motor neurons analyzed. On average, 3 motor neurons were analyzed for soma size, dendrite width, and synaptic density.

### Immunoprecipitation of ribosome-mRNA complexes

Chat-Cre;RiboTag mice, in which Cre-dependent HA-tagged Rpl22 expression occurs in Chat^+^ cells (43), were used to enrich ribosome-associated mRNA from motor neurons of the spinal cord. Immunoprecipitation was performed as described previously (43, 85). Briefly, flash frozen spinal cords were lysed in ice-cold 1% NP40 (Roche, 11332473001) lysis buffer (50 mg tissue/mL lysis buffer) containing DTT (1 mM, MilliporeSigma, 646563), 1***×*** protease inhibitor (MilliporeSigma, P8340), 0.4 units/μL RNasin Plus RNase inhibitor (Promega, N21155), 0.1 mg/mL cycloheximide (MilliporeSigma, C7698), and 1 mg/mL heparin (MilliporeSigma, H3393) using a Dounce homogenizer. Following removal of cell debris by centrifugation (18,000*g*, 15 minutes, 4°C), Rpl22-mRNA complexes were immunoprecipitated on ice from the cell lysate using a mouse anti-HA antibody (1 μL antibody/200 μL lysate, Covance, MMS-101R, RRID: AB_291262) and Pierce Protein A/G magnetic beads (Thermo Fisher Scientific, 88803). Anti-GAPDH antibody (Rockland Antibodies, 600–401-A33, RRID: AB_2107593) was used as a negative control for validation purposes (Figure 7B). Beads were washed 4***×*** with ice-cold high-salt buffer (50 mM Tris, 300 mM KCl, 12 mM MgCl_2_, 1% NP40, 0.5 mM DTT, 0.1 mg/mL cycloheximide), and RNA was eluted with RLT buffer (Qiagen, 74034) supplemented with 10 μL/mL β-mercaptoethanol. Immunoprecipitated RNA and whole cell lysate RNA was purified using the Qiagen RNeasy Plus Micro Kit (Qiagen, 74034).

### Western blot

Western blots were performed on spinal cord whole cell lysate and immunoprecipitated Rpl22 mRNA complexes from Chat-Cre;RiboTag mice, collected as described in the previous section. Protein concentrations were determined by Bradford assay, and lysates were denatured in Laemmli buffer at 95°C for 10 minutes. Samples were electrophoresed on a 10% SDS-PAGE gel and transferred to nitrocellulose membrane using wet transfer. Membranes were incubated for 1 hour at room temperature in blocking solution (5% nonfat milk in 0.1% Tween Tris buffer), incubated overnight at 4°C in primary antibody diluted in blocking buffer, washed 4***×*** in 0.1% Tween Tris buffer, incubated 1 hour at room temperature in peroxidase labeled secondary antibody, and washed 4***×*** in 0.1% Tween Tris buffer. ECL reagent (Bio-Rad, 1705060) was applied to membranes before visualization on an iBright FL1500 Imaging System (Invitrogen). Primary antibodies include mouse anti-HA (1:1,000, Covance, MMS-101R, RRID: AB_291262). Secondary antibodies include Peroxidase-conjugated Affinipure donkey anti–mouse IgG (H+L), (1:1,0000, Jackson ImmunoResearch, 715-035-151, RRID: AB_2340771).

### RNA-Seq

RNA-Seq was performed by GeneWiz on immunoprecipitated RNA from Chat-Cre;RiboTag mice. Following sequencing, data were trimmed for both adaptor and quality using a combination of ea-utils and Btrim (86, 87). Sequencing reads were aligned to the genome using HiSat2 (88) and counted via HTSeq (89). Quality control summary statistics were examined to identify any problematic samples (e.g., total read counts, quality and base composition profiles (± trimming), raw fastq formatted data files, aligned files (bam and text file containing sample alignment statistics), and count files (HTSeq text files). Following successful alignment, mRNA differential expression was determined and tested for significance using the Benjamini-Hochberg corrected Wald test in the R package DESeq2 (90). Failed samples were identified by visual inspection of pairs plots and removed from further analysis resulting in the following number of replicates for each sex and time point: 3-month male, 3; 3-month female, 4; 12-month male, 4; 12-month female, 5; 18-month male, 5; 18-month female, 5; and 24-month male, 5. Functional and pathway analysis was performed using IPA (QIAGEN Inc, https://www.qiagenbio-informatics.com/products/ingenuity-pathway-analysis). An Excel spreadsheet of the DEG data from this experiment is available in Ribotag MN RNAseq data in the supplemental material. The data that support the RNA-Seq findings of this study are openly available in NCBI GEO at https://www.ncbi.nlm.nih.gov/geo/ (accession no. GSE226699).

### RNAscope

In situ hybridization was performed on 10 μm–thick coronal spinal cord sections using the RNAscope Multiplex Fluorescence V2 system (Advanced Cell Diagnostics) according to the manufacturer’s instructions. Twenty double-Z probes targeting *Chat* (catalog 408731) or small proline rich protein 1a (*Sprr1a*) (catalog 426871) mRNA were synthesized by Advanced Cell Diagnostics. Sections were deparaffinized and underwent multiple ethanol washes and peroxidase blocking steps. Sections were then incubated in antigen retrieval buffer and digested by proteinase. Sections were incubated in the double-Z probe mixture at 40°C in a hybridization oven for 2 hours. Sections were then washed, and the signal was amplified using the included amplification and HRP reagents. The sections were then imaged via confocal microscopy using identical imaging parameters across all samples. Maximum intensity projections were generated and used for analysis. We performed visual inspection of each *Chat*^+^ motor neuron in each tiled image for presence of *Sprr1a* signal.

### qPCR

RNA collected by immunoprecipitation from Chat-Cre;RiboTag spinal cords was reverse transcribed with iScript Reverse Transcription Supermix (Bio-Rad, 1708891). cDNA was preamplified with SsoAdvanced PreAmp Supermix (Bio-Rad, 1725160), and qPCR was performed with iTAQ SYBR Green Supermix (Bio-Rad, 1725121) using 300 nM primers (Table 2) on a CFX Connect Real Time PCR System. Gene expression was normalized to *Gapdh* using the 2^–ΔΔCT^ method.

### Statistics

A 2-sided, unpaired Student’s *t* test, with Welch’s correction as appropriate, was used to compare 2 means. One-way ANOVA with Bonferroni’s post hoc analysis was used to compare 3 or more means. A Kolmogorov-Smirnov test was used to compare the distribution of α-motor neuron soma areas. An *n* was defined as 1 animal for all experiments, except comparisons of motor neuron soma size distribution and EM, where an *n* was defined as 1 α-motor neuron. Analyses were performed on distinct samples in all experiments except motor neuron soma size and EM, where individual samples were analyzed repeatedly. GraphPad Prism (RRID:SCR_002798) was used for statistical analyses. Data are expressed as mean ± SEM. A *P* value of less than 0.05 was considered statistically significant.

### Study approval

Breeding, housing, and experimental use of mice were performed in accordance with the NIH, Virginia Tech IACUC (protocol no. 18-148 and 18-176), and Brown University IACUC (protocol no. 19-05-0013) guidelines. Monkey studies were approved by the National Institute on Aging, Intramural Research Program’s Animal Care and Use Committee. Human tissue obtained from NIH Neurobiobank was approved by the University of Pittsburgh IRB (protocol no. REN14120157/IRB981146). Written informed consent was received prior to participation.

## Author contributions

Conceptualization, methodology, validation, writing of the original manuscript draft, reviewing, and editing were contributed by RWC and GV. Investigation was contributed by RWC, MCL and GV. Data curation and formal analysis were contributed by RWC and RES. Data visualization was contributed by RWC. Resources, funding acquisition, supervision, and project administration were contributed by GV.

## Supplementary Material

Supplemental data

Supplemental table 1

Supplemental video 1

## Figures and Tables

**Table 2:**
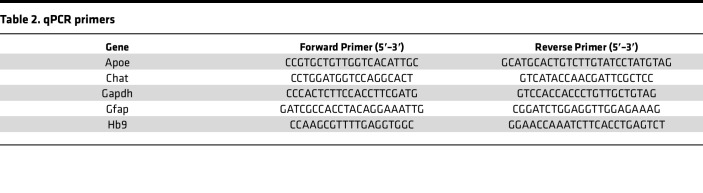
qPCR primers

**Table 1:**
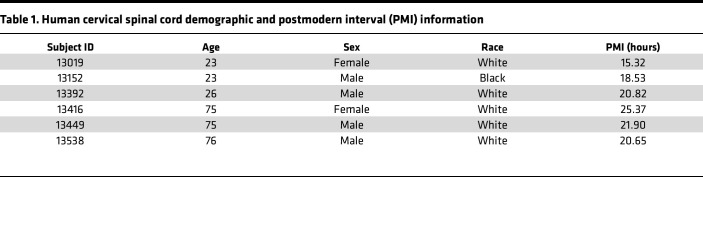
Human cervical spinal cord demographic and postmodern interval (PMI) information

## Version 1 05/08/2023

Electronic publication

## Version 2 05/18/2023

sdt1 no longer missing
