# Supplementary material for: Aging alters mechanisms underlying voluntary movements in spinal motor neurons of mice, primates, and humans
Source: JCI Insight. 2023 May 8;8(9):e168448. doi: 10.1172/jci.insight.168448 (PMC10243831; doi:10.1172/jci.insight.168448)

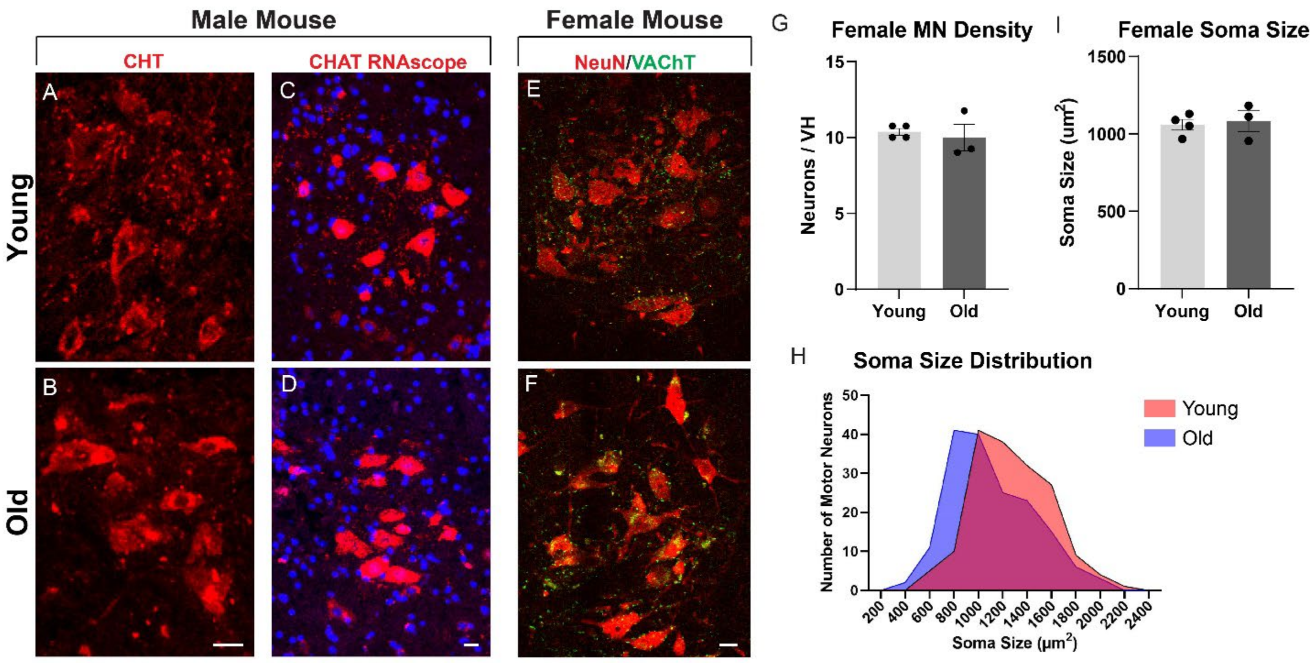

Supplemental Figure 1: Female motor neuron density and size quantification and additional representative images. (A-B) ChT IHC. (C-D) *Chat* RNAscope. (E-F) NeuN and VAChT IHC in female mice. (G) Female motor neuron density is unchanged with age. (H) Distribution of motor neuron soma sizes in young and aged male Chat-Cre;tdTomato mice. (I) Female motor neuron size is unchanged with age. All values presented as mean ± SEM; n = 3. Scale bar = 20 μm.

Supplemental Figure 2: Video of confocal z-stack images of Chat-Cre;TdTomato mouse spinal cord following tissue clearing using the PACT-CLARITY method.

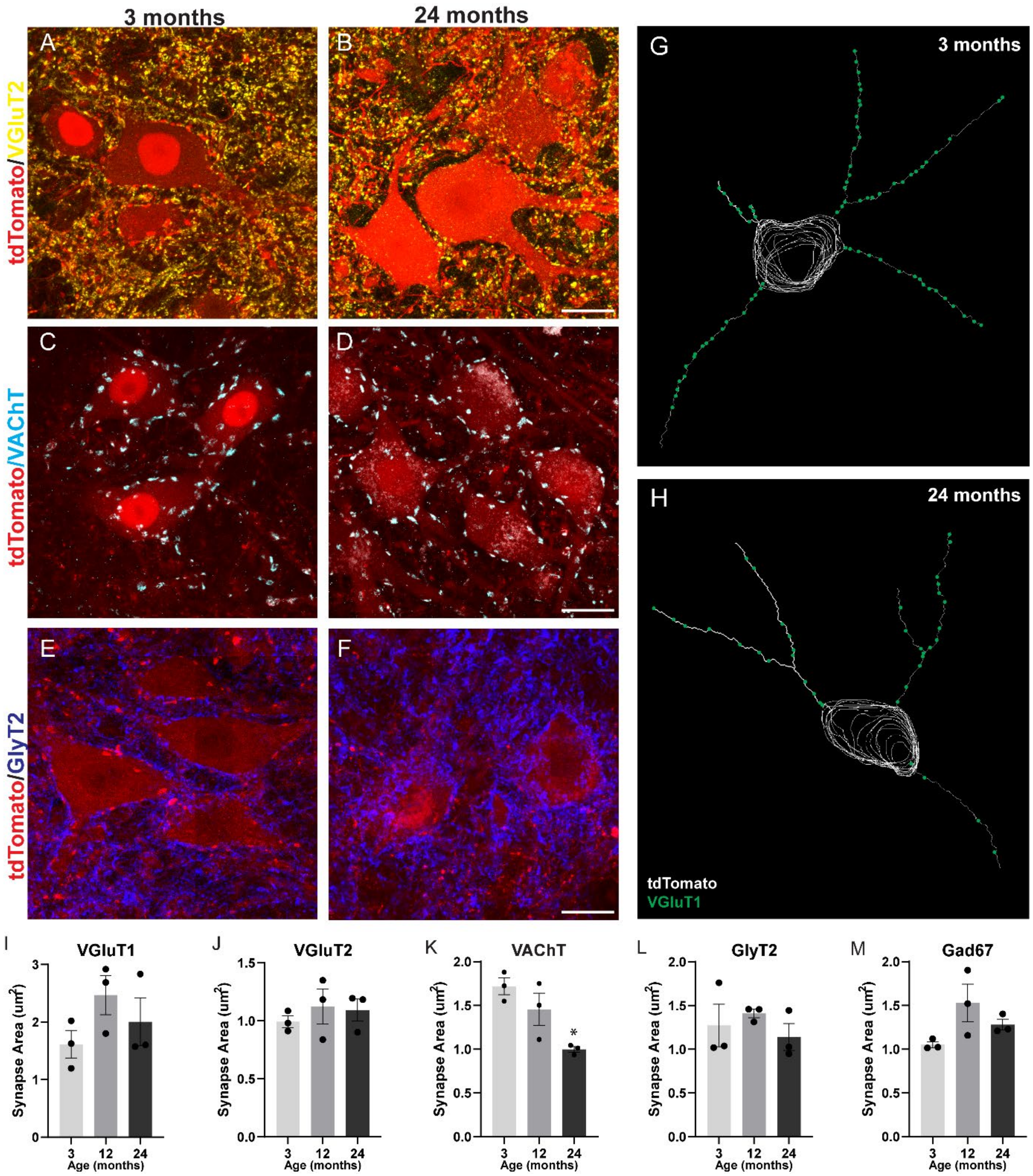

Supplemental Figure 3: Representative images of synaptic IHC on motor neurons and their average synaptic area. (A-B) VGluT2 (C-D) VAChT (E-F) GlyT2 IHC in 3- and 24-month-old Chat-Cre;tdTomato mouse spinal cord. (G-H) Representative traces showing synapses on motor neurons. (I-M) Average synapse area for each synaptic subtype. * $p < 0.05$ versus young, one-way ANOVA with Bonferroni post-hoc. All values presented as mean ± SEM; n = 3. Scale bar = 20 µm.

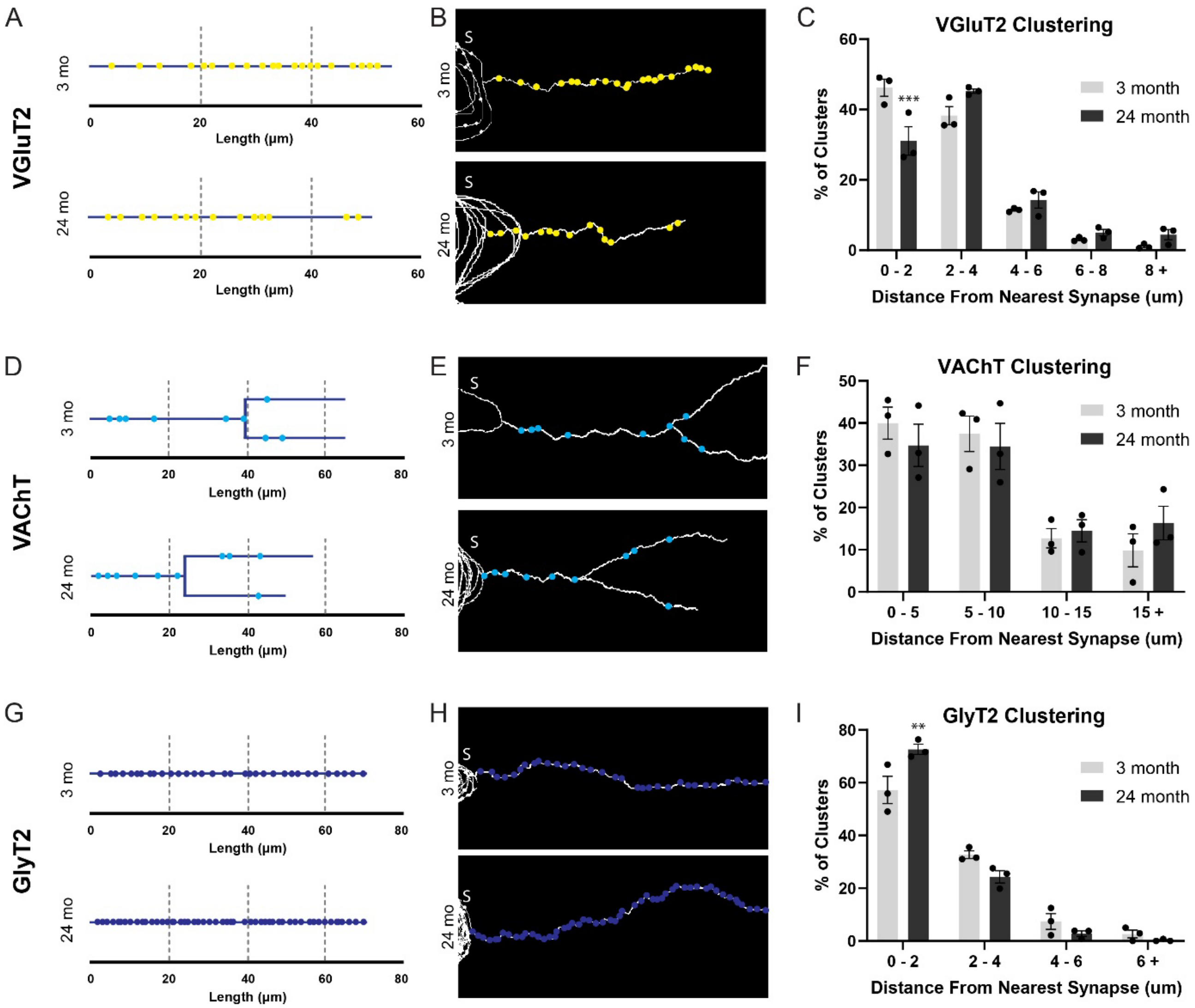

Supplemental Figure 4: Clustering of glutamatergic, cholinergic, and glycinergic synapses on motor neuron dendrites. Schematic representation of the location of VGluT2+ (A), VAChT+ (D), and GlyT2+ (G) synapses along a section of the dendritic arbor of motor neurons from 3- and 24-month-old mice. Representative neuronal traces of VGluT2+ (B), VAChT+ (E), and GlyT2+ (H) synapse locations along dendrites. 'S' labels the motor neuron soma. Quantification of the % of total dendritic synapses that have a nearest neighbor within the specified distance along the occupied dendrite for VGluT2+ (C), VAChT+ (F), and GlyT2+ (I) synapses. $^{**}p < 0.01$, $^{***}p < 0.001$, unpaired T-test. All values presented as mean ± SEM; n = 3.

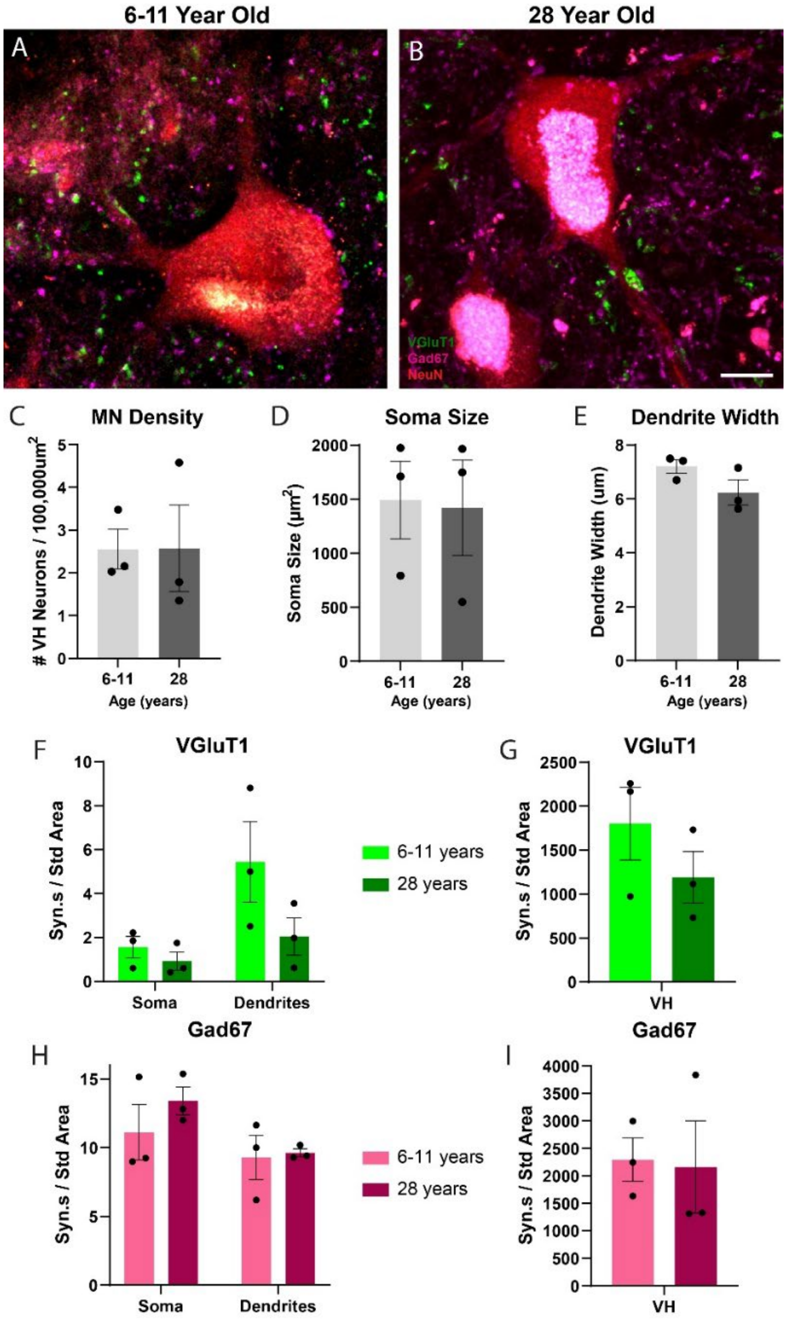

Supplemental Figure 5. Profiling of rhesus monkey motor neurons and synapses in the cervical spinal cord during aging. (A-B) Images of motor neurons (NeuN, red), glutamatergic synapses (VgluT1, green), and GABAergic synapses (Gad67, pink) on cervical spinal cord motor neurons of young and aged monkeys. (C-E) Quantifications of motor neuron density (C), soma size (D), and dendrite width (E) reveal no significant changes with age. (F-G) The number of VgluT1+ synapses on the soma and dendrites (F) and in the ventral horn (G) are unchanged, but trending toward a decrease in aged motor neurons. (H-I) Gad67+ synapses on the soma and dendrites (H) and in the ventral horn (I) are unchanged. Unpaired T-test used for all comparisons except H, where an unpaired T-test with Welch's correction was used. All values presented as mean ± SEM; n = 3. Scale bar = 20 μm.

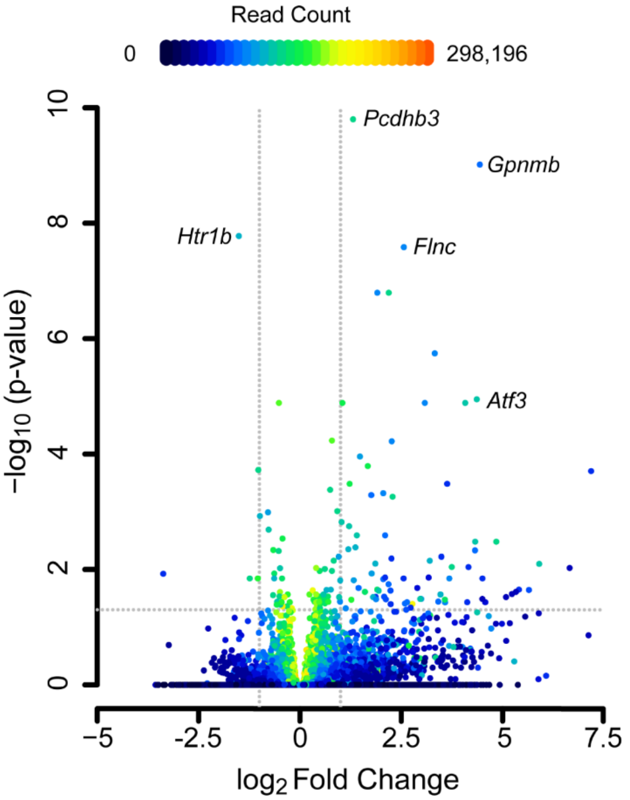

Supplemental Figure 6. Volcano plot of spinal cord motor neuron-enriched transcriptomes of 24- versus 3-month old male mice. Vertical dashed lines indicate $\log_2$ fold values of 1 and -1. Horizontal dashed line indicates p = 0.05.

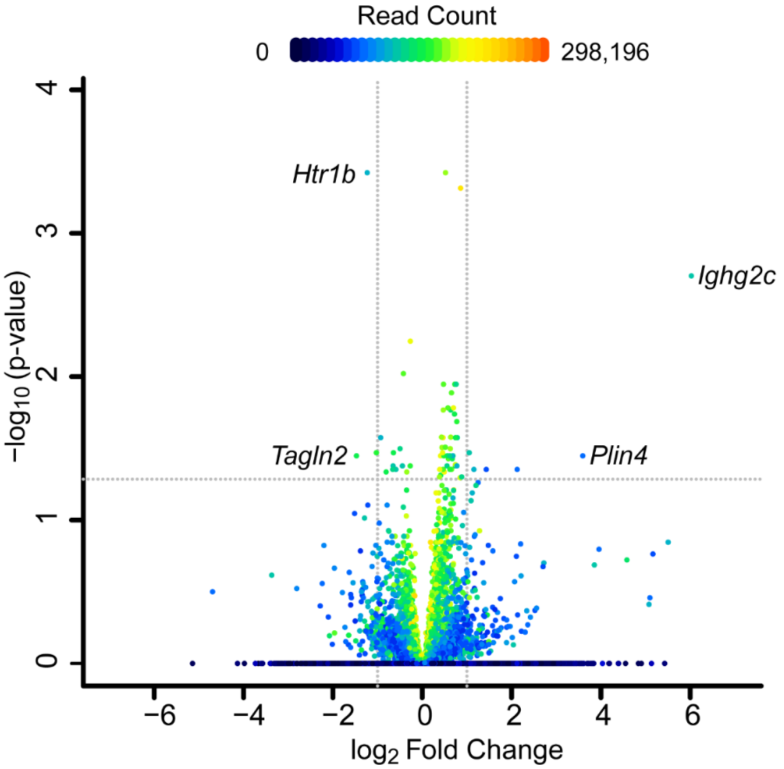

Supplemental Figure 7. Volcano plot of spinal cord motor neuron-enriched transcriptomes of 18-versus 3-month old female mice. Vertical dashed lines indicate $\log_2$ fold values of 1 and -1. Horizontal dashed line indicates p = 0.05.

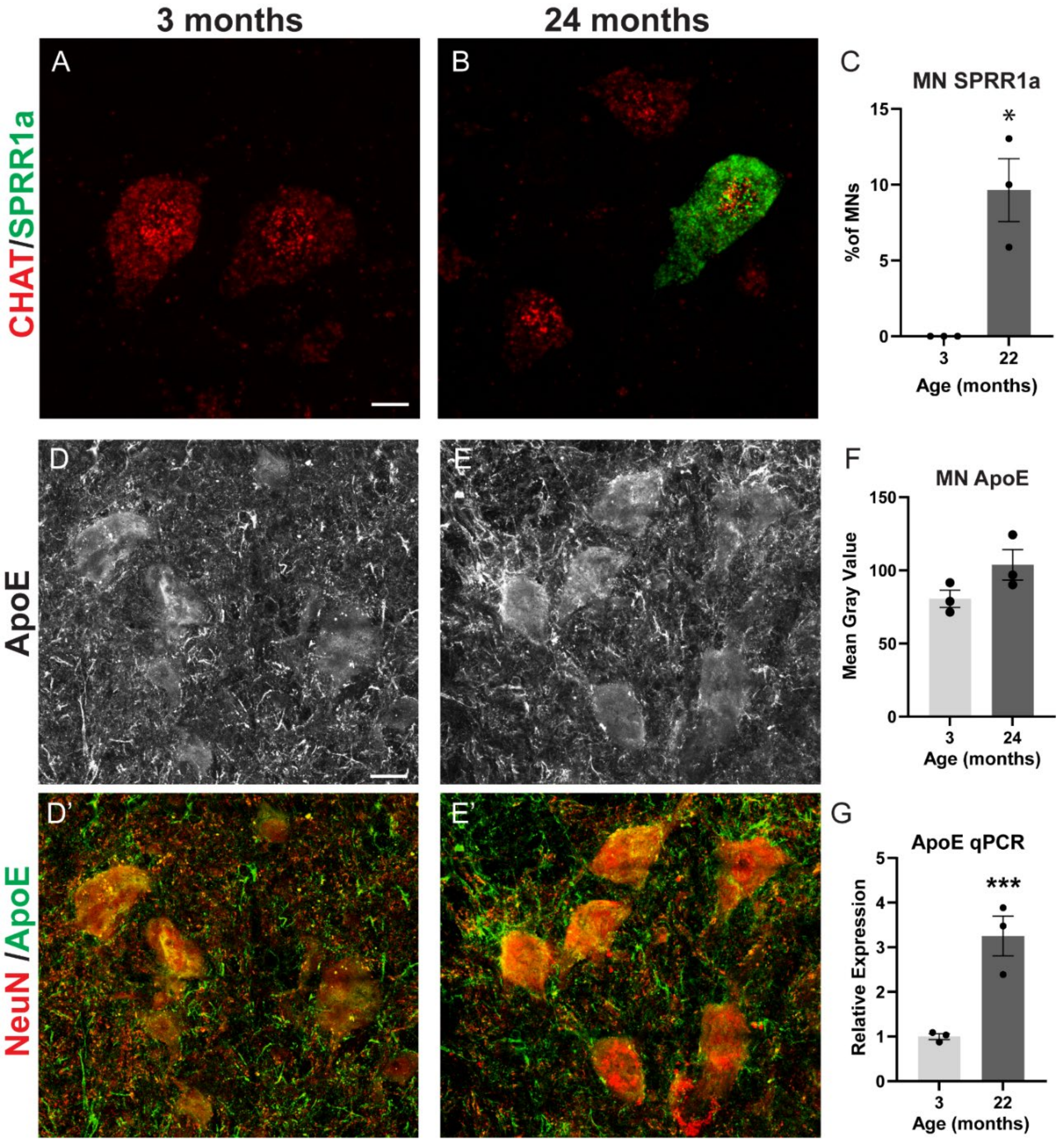

Supplemental Figure 8: RNAscope, IHC, and qPCR validation of RNA sequencing data. (A-B) Representative images of *Sprr1a* and *Chat* RNAscope showing heterogeneously *Sprr1a* upregulation among *Chat*-labeled motor neurons in 24-month-old mouse spinal cords. (C) Quantification of the percentage of *Sprr1a*-positive *Chat*-labeled motor neurons in the spinal cord. (D-E) Representative images of ApoE and NeuN IHC in mouse spinal cords. (F) Quantification of APOE fluorescence intensity shows a trend toward elevated APOE in in NeuN-labeled motor neurons of aged mice. (G) ApoE is significantly upregulated by aged motor neurons when assessed by qPCR. RNA collected by HA-IP from Ribotag; Chat Cre spinal cord. *$p < 0.05$, ***$p < 0.001$, unpaired T-test. All values presented as mean ± SEM; n = 3. Scale bar = 20 µm.

Full unedited gel for Fig. 7B

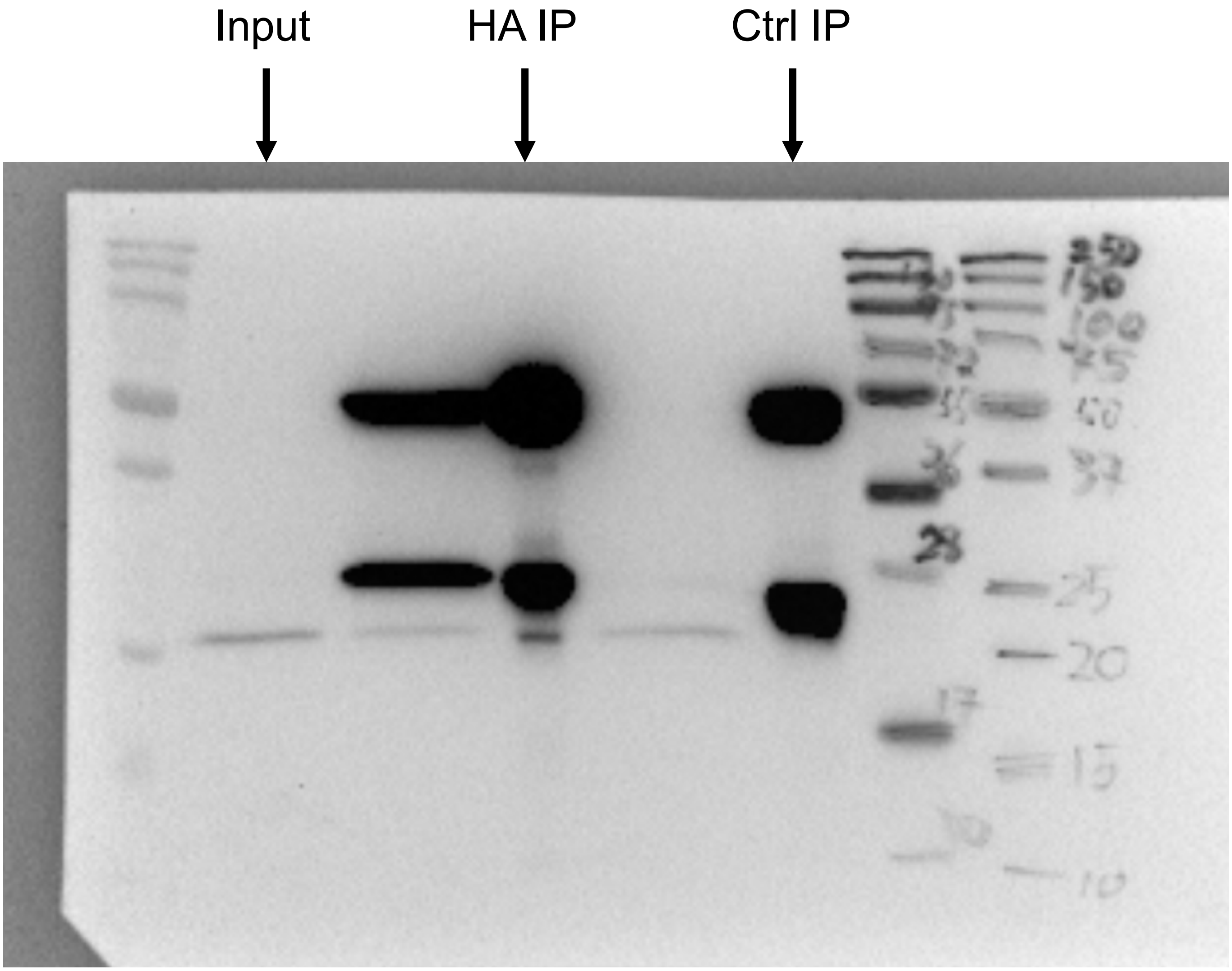

Supplement: Supplemental data [file jciinsight-8-168448-s231.pdf]
